# How Many People have Alcohol Use Disorders? Using the Harmful Dysfunction Analysis to Reconcile Prevalence Estimates in Two Community Surveys

**DOI:** 10.3389/fpsyt.2014.00010

**Published:** 2014-02-03

**Authors:** Jerome C. Wakefield, Mark F. Schmitz

**Affiliations:** ^1^Silver School of Social Work and Department of Psychiatry, School of Medicine, New York University, New York, NY, USA; ^2^School of Social Work, Temple University, Philadelphia, PA, USA

**Keywords:** alcohol use disorder, alcohol dependence, addiction, validity of diagnosis, harmful dysfunction, diagnostic criteria, psychiatric epidemiology

## Abstract

Community prevalence rates of alcohol use disorders (AUDs) provided by epidemiological studies using DSM-based diagnostic criteria pose several challenges: the rates appear implausibly high to many epidemiologists; they do not converge across similar studies; and, due to low service utilization by those diagnosed as disordered, they yield estimates of unmet need for services so high that credibility for planning purposes is jeopardized. For example, two early community studies using DSM diagnostic criteria, the Epidemiologic Catchment Area Study (ECA) and the National Comorbidity Survey (NCS), yielded lifetime AUD prevalence rates of 14 and 24%, respectively, with NCS unmet need for services 19% of the entire population. Attempts to address these challenges by adding clinical significance requirements to diagnostic criteria have proven unsuccessful. Hypothesizing that these challenges are due to high rates of false-positive diagnoses of problem drinking as AUDs, we test an alternative approach. We use the harmful dysfunction (HD) analysis of the concept of mental disorder as a guide to construct more valid criteria within the framework of the standard out-of-control model of AUD. The proposed HD criteria require harm and dysfunction, where harm can be any negative social, personal, or physical outcome, and dysfunction requires either withdrawal symptoms or inability to stop drinking. Using HD criteria, ECA and NCS lifetime prevalences converge to much-reduced rates of 6 and 6.8%, respectively. Due to higher service utilization rates, NCS lifetime unmet need is reduced to 3.4%. Service use and duration comparisons suggest that HD criteria possess increased diagnostic validity. Moreover, HD criteria eliminate 90% of transient teenage drinking from disorder status. The HD version of the out-of-control model thus potentially resolves the three classic prevalence challenges while offering a more rigorous approach to distinguishing AUDs from problematic drinking.

## Introduction and Conceptual Background

In this paper, we provide a novel reanalysis of prevalence rates for alcohol use disorder (AUD) in two major epidemiological surveys. First, in a lengthier-than-usual conceptual introduction, we offer a rationale for rethinking standard DSM-type AUD diagnostic criteria. Then, in “Materials and Methods,” systematically applying the harmful dysfunction (HD) analysis of mental disorder (1) to AUD diagnosis for the first time, we use items available in the two surveys to formulate more conceptually valid AUD diagnostic criteria that better identify dysfunction and harm. We then use the HD-derived criteria to recalculate AUD prevalence rates in the surveys, comparing the results to the prevalences yielded by the DSM-based criteria originally used in the studies, and also to the prevalences yielded by the new DSM-5 criteria.

We evaluate the HD and DSM criteria sets using a variety of validity tests. Some of the validity tests use standard validators, such as episode duration and service use, whereas others are more novel. For example, we examine the degree to which each criteria set addresses the longstanding puzzle of divergent prevalence rates of AUD across surveys, a problem tackled in classic papers by Regier et al. (2) and Narrow et al. (3) but which they failed to resolve. We also examine whether the HD analysis might explain the puzzlingly high rate of spontaneous remission among adolescents with apparent AUD, a finding often cited by those who argue that addiction is not really a disorder at all but a normal choice process (4, 5). Additionally, we use the HD analysis to provide estimates of unmet need for treatment of AUD that are dramatically different from standard estimates and address the paradox of enormous rates of apparent AUD but without any felt need for treatment.

### The puzzle of high and varying prevalence rates of alcohol use disorders in community epidemiological studies

Epidemiological studies of community prevalence of AUDs attempt to answer the question: how many people suffer from an AUD during their lifetime (lifetime prevalence) or during a given year (1-year prevalence)? The answer to the prevalence question has major implications for theories of etiology, research, and public policy.

The usefulness of such prevalence estimates depends on how validly the diagnostic criteria identify AUD. However, AUD prevalence estimates yielded by major DSM-based epidemiological surveys indicate rates of untreated disorder that many epidemiologists find implausibly high and that reveal puzzling disparities across studies with broadly similar methodologies. In particular, the first two large DSM-based community epidemiological surveys, the epidemiologic catchment area study [ECA (6)] and National Comorbidity Survey [NCS (7)] used similar methods and collected data within a decade of each other in the early 1980s and early 1990s, yet yielded lifetime prevalence of AUDs in the American population of 14 and 24%, respectively.

The more recent National Epidemiologic Survey on Alcohol and Related Conditions [NESARC (8, 9)], the largest (*N* = 43,098) cross-sectional study to date, with data collected only a decade after the NCS, reported a lifetime DSM-IV (10) AUD rate of 30%, substantially higher than the previous studies. Edwards et al. (11) recently assessed lifetime DSM-IV and DSM-5 AUD prevalence in a different data set and found essentially the same prevalence rate of 31% for DSM-IV and 32% for DSM-5 AUD.

Moreover, the lifetime DSM AUD prevalences derived from such cross-sectional studies are likely substantial underestimates due to respondents’ failure to recall symptoms. DSM AUD prevalence estimates increase dramatically in longitudinal studies in which respondents are assessed periodically for recent disorder. In the Dunedin longitudinal study of a representative New Zealand community sample, the prevalence of alcohol dependence alone (not including alcohol abuse) during one or more of four sampled 1-year periods between the ages of 18 and 32 was 32% (12). This estimate did not include those who had symptoms only during the other 11 1-year periods between ages 18 and 32, those who qualified for diagnosis only before age 18 or after age 32, and those who had alcohol abuse without dependence. Based on the abuse/dependence ratio in other studies, one can project that the Dunedin lifetime DSM-IV AUD prevalence including both dependence and abuse – or equivalently applying the new single DSM-5 AUD category – would be well over 50% of the population in just the four sampled 1-year periods. These rates are difficult to square with current claims that AUD is a brain disease (13–15).

An alternative view is that many of the diagnosed individuals in epidemiological studies do not in fact have a disorder of alcohol use at all but rather are heavy or problem drinkers for a period of time. Of NESARC AUDs, 72% reported just one lifetime episode, with a mean duration for single-episode cases of 2.7 years for abuse and 3.4 years for dependence. Other studies support high and rapid remission rates for AUDs rather than the chronic deteriorating course often predicted (16). Moreover, the rates at which diagnosed individuals seek help are extremely low. Lifetime NESARC dependence and abuse cases sought some form of service only 24 and 7% of the time, respectively. The revelation that, contrary to the “disease” model of alcoholism, large numbers of heavy drinkers manage to stop drinking heavily without therapeutic support (17) suggests a non-disorder interpretation, thus that many DSM diagnoses of AUD may be invalid if the diagnoses are intended to identify a psychiatric disorder of impaired deliberation or motivation in choosing to drink.

### Validity concerns about DSM diagnostic criteria

The ECA, NCS, and NESARC used DSM-III, DSM-III-R, and DSM-IV diagnostic criteria, respectively. It is not difficult to see why DSM AUD criteria might be invalid and give rise to inflated AUD prevalence estimates. The DSM system through its various editions has included a confusing mélange of symptoms, conceptualizations, and categories for AUD, and the criteria for AUD have often consisted primarily of harmful or socially undesirable effects of alcohol use that can equally be present in non-disordered heavy use of alcohol. Moreover, some DSM indicators of dependence have been equally indicative of strong desire or habit within a drinking-accepting environmental context. The general problem with DSM criteria has been the use of criteria that lack adequate specificity for identifying cases in which it can be inferred that there is an underlying dysfunction of alcohol desire, as opposed to negative effects of alcohol or indicators of strong desire to drink alcohol. A systematic critique of DSM criteria is beyond the scope of this article, but detailed specifications of DSM-III (18), DSM-III-R (19), and DSM-5 (20) AUD diagnostic criteria and how they were translated into ECA and NCS diagnostic criteria are provided in the Tables A1, A2, and A5 in Appendix, respectively.

Whereas DSM-III through to DSM-IV distinguished alcohol abuse and dependence, the abuse category was eliminated in DSM-5. Yet the abuse criteria (except for legal difficulties) were incorporated in DSM-5 into a generic AUD category along with dependence criteria and a new “craving” criterion, with a diagnostic threshold lowered to 2 symptoms out of 11 from the previous three symptoms out of seven. Thus, an individual can now be diagnosed with AUD on the basis of symptoms that are very weak indicators of dysfunction, such as the former abuse symptoms of arguing with family members over alcohol use and driving under the influence of alcohol.

### Failure of the Regier et al. (1998) and Narrow et al. (2002) attempts to improve AUD diagnostic validity

Based on the premise that implausibly high community disorder rates were due to the use of overly broad DSM diagnostic criteria, two now-classic epidemiological analyses by Regier et al. (2) and Narrow et al. (3) attempted to resolve the issue of high and divergent prevalence rates emerging from the ECA and NCS epidemiological surveys. Their reanalyses addressed a broad range of disorders, but we limit the present discussion to AUDs.

Regier et al. (2) considered only alcohol dependence, not alcohol abuse. After limiting the two studies to a common age range of 18–54 years old, the ECA and NCS 1-year prevalences (generally considered more valid than lifetime prevalences in cross-sectional studies relying on memory) were a divergent 4.1 and 7.4%, respectively, with lifetime prevalences similarly divergent at 8.6 and 14.9%, respectively. Regier et al. performed a series of corrections to the two studies’ data sets to try to bring the divergent 1-year rates more into harmony. These included limiting both samples to those with certain race or geographic demographic characteristics that yielded adequate subsample sizes in both studies to minimize variance, weighting frequencies in both studies to match age and gender frequencies in the 1990 Census, and applying similar DSM-III-based diagnostic criteria with as closely similar wording as possible across studies. Despite these corrections, the ECA and NCS alcohol dependence prevalence rates remained high and divergent at 4.6 and 8.3%, respectively.

Regier et al. suggested that the problem could lie instead in inflated diagnostic rates due to invalid DSM criteria yielding false-positive diagnoses: “The obvious question is whether each of the final groups contains subjects with valid clinical diagnoses or if either or both have a high proportion of false-positive responses. … Based on the high prevalence rates identified in both the ECA and the NCS, it is reasonable to hypothesize that some syndromes in the community … do not represent true psychopathologic disorders” (pp. 112, 114). The implication was that changes in diagnostic criteria to improve validity were required.

Extending Regier et al.’s work, Narrow et al. (3) examined all AUD including dependence and abuse, but only 1-year disorder. They attempted to increase diagnostic validity by imposing a clinical significance criterion, requiring service use or interference with life a lot, on all ECA and NCS diagnoses. Noting that NCS prevalence rates are generally higher than ECA rates, Narrow et al. also used the questionable strategy of combining the primary ECA data with a second wave of ECA data collection a year after the first, in which newly emergent symptoms as well as newly recalled symptoms from the past were reported. They then compared the cumulative two-wave ECA data to the NCS one-wave data, thus increasing the overall ECA AUD prevalence rate to be closer to the higher NCS rate (9.1 versus 9.9%, respectively). However, when they applied the clinical significance requirement to increase validity, the resulting 1-year AUD rates diverged significantly, with final rates of 8.9 and 6.5%, respectively.

The Narrow et al. reanalyses thus failed to establish convergence for AUD. They also suffered from several problems. First, there was not a persuasive rationale for comparing two-wave ECA data to one-wave NCS data, except the *ad hoc* desire to force rates to converge. Second, the appropriateness of using service contact as a clinical significance diagnostic criterion is questionable, because it undermines the point of a community study (21). Third, clinical significance criteria generally have been found to have little power to distinguish disorder from non-disordered distress (22, 23). Finally, divergent lifetime estimates were not addressed.

The Narrow et al. results triggered a debate that continues to this day over whether cases eliminated from epidemiological disorder diagnoses by clinical significance criteria are mild disorders or not disorders at all (24–28). Kessler et al. (24) accused Narrow et al. (3) of “an attempt to declare that mild cases do not exist” (p. 1118). Regier et al. (28) responded that the goal is to increase homogeneity and therefore validity: “Our objective is to define increasingly homogenous diagnostic groups with greater predictive validity with respect to both prevention and treatment response” (p. 1059). Given that disorders can be mild and that non-disordered heavy drinking can be quite harmful, an approach to increasing validity and homogeneity other than by a clinical significance threshold appears desirable.

### The harmful dysfunction analysis of the concept of mental disorder

The study reported here attempts to improve the conceptual validity (i.e., the disorder/non-disorder differentiation) of AUD criteria by directly altering the diagnostic criteria rather than by adding clinical significance criteria. Our attempt is guided by the HD analysis of the concept of mental disorder (1, 29–34). The DSM-5 definition of mental disorder asserts that a psychological disturbance and the consequent distress and role impairment is a disorder only when it “reflects a dysfunction in the psychological, biological, or developmental processes underlying mental functioning” [(20), p. 20]. The HD analysis elaborates the definition’s concept of dysfunction as failure of some psychological mechanism to perform its naturally selected biological function. Dysfunction in this sense is *not* equivalent to “dysfunction” in the sense of failing to function effectively in various social roles such as in occupational or marital roles (as in a “dysfunctional marriage” or when one is “dysfunctional at work”). Such failures are a form of harm, but they often exist in non-disordered conditions and are not dysfunctions in the definition’s intended sense of the failure of some underlying psychological mechanism. Given the frequent harmfulness of alcohol, the dysfunction requirement is critical to the distinction between disordered and non-disordered drinking.

The judgment that harm is being caused by a dysfunction is often highly inferential and fallible given our limited knowledge, yet nonetheless implicit in all disorder diagnoses. Often, inferences to underlying dysfunction are based on the context of the symptoms (35), yet contextual information is lacking in many AUD criteria (e.g., did you drink more than intended because of social pressure, because the drinking itself made you more relaxed about your goals, because you wanted a more intense high, or because you no longer felt in control of your choice?). The DSM-5’s and HD analysis’s dysfunction requirement underscores that social deviance and conflicts between the individual and society, although often warranting intervention, are not mental disorders unless they are due to underlying dysfunctions. This provides a conceptual “firewall” between sheer social control – such as intervention to stop people from driving while intoxicated or to prevent alcohol-facilitated foolish sexual choices – and medically necessitated psychiatric treatment for disorder, in an area in which moral and psychiatric judgments may easily get confusingly mixed together.

### The concept of mental disorder and the loss-of-control model of alcohol use dysfunction

Given the lack of gold standards for dysfunction or, for that matter, significant harm, the application of the HD analysis to the domain of alcohol disorders depends on many decisions and judgments that are disputable. Thus, there is no one unique HD “solution” to how to diagnose alcohol disorders. Moreover, standard epidemiological studies do not necessarily ask questions in the way most favorable for an HD analysis, so any reanalysis has to be approximate and make conceptual compromises.

In this initial attempt to construct HD-inspired diagnostic criteria for AUD, we do not propose a new conception of the relevant dysfunction. Rather, we provisionally accept the standard view that the dysfunction in AUD involves a “dependence syndrome” in which there is impairment of deliberation or motivation that entails a pathological degree of loss-of-control over alcohol drinking (36–41). The loss-of-control account was the basis for both the ICD-10 and DSM-III-R and DSM-IV approaches (42, 43).

We attempt to be more rigorous about which criteria indicate harm, which indicate dysfunction, and which indicate neither. Edwards and Gross were explicit in their “biaxial” model that the dependence syndrome as dysfunction must be distinguished from all the many serious harms, which themselves of course may warrant medical attention, that alcohol use can cause: “A person may, for example, develop cirrhosis, lose his job, crash his car, or break up his marriage through his drinking without suffering from the dependence syndrome …. [T]he diagnosis of dependence … should be made in relation to the primary symptoms … and not by reference to the secondary damage” [(37), pp. 1060–1]. We believe that the most plausible interpretation of the biaxial conception was ignored when DSM separated dependence, a presumptive dysfunction, from abuse, a presumptive harm; as DSM-5’s definition of mental disorder indicates, to be a disorder a condition must possess both dysfunction and harm, so neither is a disorder by itself. To this extent, the DSM-5’s change to one AUD disorder combining dependence and abuse criteria makes sense. However, the DSM-5’s threshold for diagnosis of any 2 out of 11 symptoms undermines the validity of diagnosis by not requiring that both dysfunction and harm are present. The power of the HD approach to limit false-positives and improve validity lies in distinguishing harm indicators from indicators of loss-of-control dysfunction.

An alternative option would be to abandon the loss-of-control view and embrace some alternative framework for understanding dysfunction in AUD. However, no competing conception of the possible dysfunction underlying AUDs conceived as psychiatric disorders is as well-developed as the out-of-control account at this time. Brain-disorder accounts of substance use disorders, while varying in the specific brain mechanisms that are proposed as dysfunctional, are largely aimed at providing a deeper explanation of the loss-of-control phenomenon and so fall within the same domain.

In the recent literature, there are many who reject the interpretation of alcoholism as a disorder caused by a psychological dysfunction of deliberation or motivation. They propose instead that behavior labeled addiction is the result of normal choice mechanisms. The account of addiction as disorder has been particularly challenged by empirical data on non-clinical community patterns of substance use showing high rates of spontaneous quitting even after prolonged heavy use among those qualifying for substance use disorder diagnoses (44–48). Moreover, in some psychiatric accounts, the “out-of-control” description of the hypothesized deliberative or motivational dysfunction has been implausibly exaggerated into a total lack of control rather than a degree of impairment and even, in some early descriptions, into an almost inevitable descent into madness and death, which does not at all fit the data and has garnered further skepticism. There has consequently been enormous interest in models of decision making that might explain apparent addictions as resulting from normal choice processes rather than dysfunction (5, 49–56). These authors point out that patterns of drinking that have been called compulsive are predicted by well-established choice models and that this approach is supported by much available data. These alternative approaches to alcoholism predict that many who meet the presumed criteria for loss-of-control (usually interpreted as dependence, not abuse) will be able to quit drinking without professional help, contrary to some standard “pathology” views.

We explore a different approach here to understanding the provocative finding that so many “out-of-control drinkers” don’t seek help and manage to quit drinking. We suggest that the hypothesized loss-of-control AUD dysfunction has not been translated rigorously enough into diagnostic criteria, yielding high false-positive vulnerability. Thus, the true AUDs that do exist have been obscured in epidemiological data by a tidal wave of non-cases that, although they have various alcohol problems and symptoms, are misclassified as AUDs understood as alcohol addiction. According to this view, the “normal choice” accounts may well explain many or even most cases classified as AUDs in the community epidemiological literature, but these cases have been miscategorized as disorders due to invalid diagnostic criteria.

### Aims of the study

The present study uses the HD analysis to reformulate AUD diagnostic criteria in an attempt to more validly capture the “out-of-control” model of AUD. In this initial test of the viability of this approach, we restrict our analysis to the ECA and NCS studies. In evaluating the results, we adopt the same four criteria as Narrow et al.: lower AUD prevalence; converging ECA and NCS AUD prevalence estimates; greater validity of criteria as indicated by AUD cases manifesting standard validators; and increased concordance between diagnosis and treatment, thus more meaningful estimates of unmet need for AUD services. We compare the HD analysis to traditional DSM criteria and to DSM-5 criteria.

## Materials and Methods

### Sample and measures

Two datasets were used in these analyses. The NCS (7) is a community-based epidemiological survey administered in face-to-face interviews between September 14, 1990 and February 6, 1992, to 8,098 persons aged 15–54-years who are representative of the US population. The sample used here consists of all adults aged 18–54 (*N* = 7,599). The ECA (6) interviewed respondents aged 18–98 (*N* = 19,182) at five sampled sites (Baltimore, MD, USA; Durham, NC, USA; Los Angeles, CA, USA; St. Louis, MO, USA; and New Haven, CT, USA) face-to-face between 1980 and 1985. Our analytic sample includes only adults aged 18–54 (*N* = 11,092). Data were weighted to account for selection and non-response effects, and to match age, sex, and race distributions in the US Census, in order to provide nationally representative estimates.

### HD alcohol use disorder diagnostic criteria

For the purpose of our HD analysis of AUD, we assumed, consistent with the majority of the field’s literature over the past half-century, that the psychiatric disorder category of AUD refers to a disorder in which something has gone wrong with the functioning of the individual’s systems of deliberation, motivation, and decision making, when it comes to partaking of alcohol (57). We also accepted the standard view that this change in motivational structure can come about either due to physiological changes (e.g., withdrawal symptoms when one stops drinking, drinking to prevent withdrawal symptoms, inability to function normally without alcohol use) or psychological reasons (e.g., inability to stop or cut down drinking despite wanting to do so, craving for alcohol). We proceeded similarly to DSM-5, defining one disorder that includes all conditions deemed AUDs, rather than two (dependence and abuse) as in DSM-IV.

We created three versions of the diagnostic criteria for HD AUD. All were shaped by the HD conceptualization, but each drew on a different set of questions. The “HD/ECA” version is formulated within the constraints imposed by the questions available in the ECA, as shown in Table A3 in Appendix. The “HD/NCS” version is formulated using the somewhat broader NCS question set, as shown in Table A4 in Appendix. These HD formulations allowed us to test within each study for differences between standard and HD prevalence rates. The third “HD/NCS (ECA comparable)” version, also shown in Table A4 in Appendix, was based on NCS questions but limited so as to be optimally comparable to the HD/ECA criteria. This formulation allowed us to test whether increasing the comparability of HD criteria across studies yielded more convergent ECA and NCS prevalence rates.

To create these HD criteria sets, we categorized a study’s symptom questions into the two broad HD components of dysfunction and harm based on consensus after extensive discussion. Symptoms that fit neither category of dysfunction or harm (e.g., tolerance, habitual use of alcohol) are not shown and were ignored in our HD formulation. Because virtually all diagnostic criteria for AUD can have false-positive instances in which there is no disorder but only normal intense desire, judgments of how to categorize symptoms inevitably involved subjective judgments about optimally balancing the elimination of false-positive diagnoses against the avoidance of false negative diagnoses, and the judgments of others might diverge from ours.

We made the HD AUD as broad as possible by requiring that any one or more dysfunction criteria and any one or more harm criteria be met for diagnosis. The HD/ECA dysfunction questions included: withdrawal symptoms (shakes, fits, DTs, hallucinations); need a drink before breakfast; could not do your ordinary daily work well unless you had had something to drink; and wanted to stop drinking but couldn’t. The HD/NCS dysfunction criteria included: withdrawal symptoms when stopping drinking; use of alcohol to make withdrawal symptoms go away; either persistently wanting to stop or cut down or actually trying to cut down or stop drinking alcohol, but being unable to do so; and feeling such a strong desire for alcohol that one could not resist it. The set of symptoms indicating harm were extensive in both studies (Tables A3 and A4 in Appendix).

We formulated the best HD criteria for each study, allowing criteria to differ across studies. The literature tends to emphasize that relatively small differences in criteria can sometimes cause rather large differences in prevalence rates. Consequently, previous attempts to achieve convergent AUD rates attempted to homogenize diagnostic criteria across studies (2, 3). In contrast, our HD-type criteria diverged considerably across studies in both content and wording of the specific dysfunction and harm items, as the appendix tables indicate. We hypothesized that if there is a cogent underlying AUD construct, and if diagnostic criteria selected from each study validly reflect this construct, then varying syndromal definitions should approximate to the same construct and yield convergent results.

A potentially controversial decision was not to include as a dysfunction indicator what is known as the “larger/longer” question, which asks whether the respondent drinks larger amounts or longer than intended. Larger/longer is sometimes labeled an “out-of-control” question (11) but we did not think this question as currently formulated indicates dysfunction with adequate specificity. The question overestimates the rationality of human agents by assuming that prior judgments about what is best, which may be shaped by social expectations, normally control later behavior. Drinking more than intended is commonly due to social pressure rather than compulsion; being the only one to stop drinking in a group can be quite difficult. Moreover, the larger/longer question refers to behavior that occurs during an episode of drinking, after one starts to drink. Thus, the disinhibiting effects of alcohol itself on one’s resolve rather than a motivational disorder about needing to drink alcohol can be responsible for drinking more than intended. More specificity in the question might make it usable as a dysfunction indicator in the future.

Another controversial judgment was that continuing to use alcohol despite knowledge of negative consequences – for example, threats to health or family conflicts over use – does not constitute adequate evidence to infer a dysfunction. Many people drink against medical advice, and we concluded that this is a problem but not *prima facie* specifically a dysfunction. The fact that rats may continue to engage in alcohol use despite foot shocks has been cited as a dependence indicator (58), but this observation begs the question between strongly preferred use and loss-of-control. For example, the classic demonstration of the normal curiosity motive is that chimps will undergo electric shocks to look out of a window, but no one concludes they have a curiosity addiction.

The HD analysis maintains that a dysfunction is only a disorder if it causes harm. However, there can be disagreement about precisely what forms of harm should be allowable. For example, the DSM relies heavily on social role impairment to fulfill the “harm” criterion, whereas the ICD aspires to separate disorder diagnosis from evaluation of role impairment due to the heavy cultural loading of role impairment. Some argue that when a dysfunction’s harm is due solely to social disapproval or stigma, labeling the condition as a disorder illegitimately pathologizes anomalous variation that would be benign in a context of social tolerance (59–62). However, in this initial test of the HD approach, we included all available harm items of whatever nature within our HD criteria for each data set. Given that we were predicting a substantial reduction in prevalence, we did not want to construct the “harm” criterion in a way that could be seen as biasing the result toward our predicted lower prevalence. We thus construed “harm” in the broadest possible terms, including such socially anchored harms as family arguments, to allow for the most challenging test of our prediction.

### Diagnostic criteria for comparison rates of alcohol use disorder: ECA, NCS, DSM-5, and Narrow et al.

We used the standard AUD variables from the NCS and ECA, which include both dependence and abuse (Tables A3 and A4 in Appendix), to calculate standard lifetime and 1-year prevalence rates to compare to the HD rates.

Lifetime and 1-year disorder diagnoses in both the ECA and the NCS were calculated using a “broad” approach that required fulfilling diagnostic criteria with symptoms experienced at some point, but not necessarily during the same period of time as required by the DSM (1-year disorder required at least one symptom in the last year). To evaluate the effects of moving from broad to narrow 1-year definitions where all symptoms must occur in the last year, we also calculated the “narrow” version of NCS 1-year AUD.

We also created lifetime and 1-year NCS DSM-5 prevalence estimates based on an approximation to the new DSM-5 diagnostic criteria, requiring at least 2 out of 11 symptoms. (The ECA contained insufficient comparable questions to make an ECA DSM-5 version practical.) The NCS questions used for the DSM-5 AUD diagnostic criteria are indicated in Table A5 in Appendix. One-year DSM-5 AUD was calculated using the narrow approach; at least two symptoms qualifying the individual for diagnosis must have occurred within the year prior to the interview. Finally, we also cite for comparative purposes the prevalence rates derived from Narrow et al.’s (3) reanalysis of the ECA and NCS, described above.

#### Use of DSM combined dependence and abuse versus dependence-only in AUD comparison rates

DSM-III inaugurated the distinction between dependence and abuse, which lasted through to DSM-IV and has been eliminated in DSM-5. We compare HD prevalence to standardly cited AUD prevalence rates, which in the ECA and NCS as well as most published studies of AUD, including all the major studies evaluating DSM-5 AUD criteria, are based on combined dependence and abuse. On its face, “abusing” alcohol does not appear to imply disorder, so one might wonder why we include abuse in the primary AUD analyses rather than just focusing on dependence cases which seem more tailored to the out-of-control model. There are three reasons. First, despite the seeming semantic inconsistency, DSM-III and subsequent DSM editions have classified abuse as an AUD, and all major epidemiological surveys have followed suit in reporting their results. To take one example, in Sussman et al.’s (63) attempt to estimate overall addiction rates, they note, “Both drug ‘abuse’ and ‘dependence’ were considered as ‘addicted’ in the table and in our calculations” (p. 5). The classification of abuse as a true AUD generally has been justified by claims that it is a mild form of dependence or that it is a prodromal condition increasing risk for dependence. Second, the new DSM-5 approach has eliminated the dependence/abuse distinction and reflects a “dimensional” view that there is no in-principle separation of abuse from dependence, which are interpreted to represent milder and more severe points on a continuum of disorder. To pursue legitimate comparisons to DSM-5, we needed to include DSM abuse cases from earlier criteria sets. Third, we needed to include DSM abuse within our primary comparisons because those with DSM abuse can in principle satisfy HD (as well as DSM-5) diagnostic criteria. This is because we define HD AUD to include any individual with one form of dysfunction and one harm. Thus, an individual might meet DSM abuse criteria based on one harm, and also have only one dependence symptom (e.g., an individual might have withdrawal symptoms and also drive under the influence but have no other symptoms), and thus not qualify for dependence diagnosis, and yet meet HD AUD based on a harm and a dysfunction (and also meet DSM-5 criteria based on 2 out of the 11 possible abuse and dependence symptoms). Thus, restricting the comparison to dependence would have foreclosed the empirical question of whether DSM abuse cases qualify as HD cases (or, for that matter, as DSM-5 cases).

However, we were cognizant of the possible objection that it is really the criteria for DSM dependence that represented the true out-of-control construct, thus that the HD analysis appeared to be an improvement only because it was compared to the broader category of combined dependence/abuse rather than the stricter category of dependence to which it is quite similar. To address this concern, we repeated all of our primary analyses, but this time comparing HD disorder to DSM dependence rather than combined dependence/abuse. We assess whether this change in comparison category changes our conclusions in important ways.

### Validator variables

Several variables were used as validators in the analyses, all based on lifetime NCS reports. One validator was directly associated with alcohol use: mean duration of the AUD, calculated from age of onset and age of recency reports. Three validators assessed service use associated with the use of alcohol or drugs: percentage who ever saw a mental health professional about substance use; percentage who ever attended AA or NA meetings because of their substance use; percentage who ever went to a drug or alcohol outpatient clinic for help with emotions, nerves, or use of alcohol or drugs. Non-substance use comorbidity was assessed by the percentage of respondents having any lifetime mood or anxiety disorder, assessed with the standard NCS/DSM-III-R criteria based diagnostic variables.

Once we had the results of our primary analyses, we then performed several *post hoc* analyses to illuminate the meaning of our results. These included tests of HD specificity, HD sensitivity, HD item frequencies, and a comparison of HD and standard remission rates. These tests are described below in Results.

### Statistical analysis

All of the statistical analyses used Stata 12 survey estimation procedures (64), which calculate weighted coefficients (using the ECA and NCS weights) to yield national estimates, and use Taylor series linearization to calculate standard errors, adjusting for the complex sampling designs of the two surveys. Because of the overlapping nature of the different groups, independent sample *t*-tests were not performed, and significant differences are indicated by non-overlapping 95% confidence intervals presented in the results below.

## Results

Demographic characteristics of those with standard NCS AUD and those with the HD version of NCS AUD are presented in Table 1, for both lifetime and 1-year diagnoses. Differences are minimal, with 1-year HD disordered individuals tending to be slightly older and less educated than 1-year NCS AUD individuals.

**Table 1:** **Means and percentages. (95% confidence intervals) for demographic variables of lifetime and 1-year alcohol disorders, ages 18–54, *N* = 7,599**.

	Lifetime^a^		1-year^b^	
	NCS (*n* = 1,947)	HD/NCS (*n* = 550)	NCS (*n* = 793)	HD/NCS (*n* = 336)
Female (%)	31.5 (28.0, 34.9)	31.2 (25.3, 37.1)	27.8 (23.4, 32.3)	27.5 (19.3, 35.7)
Mean age	33.3 (32.6, 33.9)	34.6 (33.6, 35.6)	30.7 (29.7, 31.7)	34.1 (33.0, 35.3)
Mean years of education	12.8 (12.5, 13.0)	12.2 (11.9, 12.4)	12.4 (12.2, 12.7)	11.9 (11.6, 12.2)
White (%)	84.0 (79.6, 88.4)	80.9 (74.0, 87.7)	82.5 (76.5, 88.4)	79.8 (71.6, 88.0)

*Weighted and corrected for sampling design*.

*NCS, National Comorbidity Survey; HD, harmful dysfunction. See “Appendix” for details of diagnostic criteria*.

*Significant differences between groups are indicated by non-overlapping 95% confidence intervals*.

*^a^For each lifetime diagnostic criteria set, the symptoms could occur at any time point in the respondent’s lifetime; the symptoms did not need to occur together*.

*^b^For the 1-year NCS criteria set, at least one symptom had to appear in the year prior to the interview; in the 1-year HD diagnostic criteria set, all symptoms had to appear in the year prior to the interview*.

Lifetime ECA and NCS AUD prevalence estimates for ages 18–54 using various diagnostic criteria sets are presented in Table 2. Standard ECA and NCS criteria yield AUD prevalences of 15 and 25%, respectively. The DSM-5 criteria yield a lifetime prevalence estimate of 19.5%, significantly higher than the ECA’s but significantly lower than the NCS’s. The HD criteria yield dramatically lower lifetime prevalence estimates for both the ECA and the NCS of 6 and 6.8%, respectively, both significantly and very substantially lower than the ECA, NCS, or DSM-5 rates. This addresses the first challenge of the seemingly implausibly high prevalence rates.

**Table 2:** **Lifetime^a^ prevalence (95% confidence intervals) for alcohol use disorder (AUD) in the ECA and NCS community studies, using standard versus harmful dysfunction (HD) diagnostic criteria, and limited to common age range of 18–54**.

Community study	Diagnostic criteria used to calculate AUD prevalence rate			
	Standard ECA and NCS criteria	DSM-5 Criteria	HD/ECA and HD/NCS criteria	HD/ECA and HD/NCS (adjusted to be ECA comparable) criteria
ECA (*N* = 11,092)	15.4 (14.6, 16.1)		6.0 (5.4, 6.6)	6.0 (5.4, 6.6)
NCS (*N* = 7,599)	24.9 (23.1, 26.7)	19.5 (18.0, 21.0)	6.8 (5.9, 7.7)	5.5 (4.9, 6.1)

*Weighted and corrected for sampling design*.

*ECA, epidemiologic catchment area study; NCS, National Comorbidity Survey; HD, harmful dysfunction. See “Appendix” for details of diagnostic criteria*.

*Significant differences between groups are indicated by non-overlapping 95% confidence intervals*.

*^a^For each diagnostic criteria set, the symptoms could occur at any time point in the respondent’s lifetime; the symptoms did not need to occur together*.

For comparison purposes, we note that the reported ECA and NCS lifetime dependence prevalence rates, excluding abuse-only cases, as reported by Regier et al. (2), are 11.3 and 14.9% for the ECA and NCS, respectively. These dependence rates are still substantially above HD AUD rates.

To test whether ECA and NCS prevalences converge if similar criteria are used, we recalculated the HD/NCS criteria using the ECA comparable version. This yielded 6% for the ECA and 5.5% for the NCS, which were not significantly different. Indeed, even before this correction the ECA and NCS HD rates of 6% and 6.8%, respectively, were not different. This addressed the second challenge, of showing that with more valid diagnostic criteria, the rates across studies might converge.

Similar results were found for 1-year prevalence estimates (Table 3). The 1-year AUD estimates using the ECA and NCS standard criteria were 7.3 and 9.9%, respectively. These rates were significantly different from each other, and significantly higher than the corresponding HD analysis’s estimates for the ECA and NCS of 3.3 and 4.3%, respectfully, which were not significantly different. Recalculating the NCS prevalence using a narrow approach that required clustering of criteria lowered the rate considerably (7.0%) but still left it significantly above the HD rate, demonstrating that the reduction resulting from the HD analysis was not due to the change to narrow criteria alone but to the differences in symptom criteria for disorder. The DSM-5 prevalence estimate (9.8%) is virtually identical with the standard NCS result.

**Table 3:** **One-year ^a^prevalence (95% confidence intervals) for alcohol use disorder (AUD) in the ECA and NCS community studies, using standard versus harmful dysfunction (HD) diagnostic criteria, and limited to common age range of 18–54**.

Community study	Diagnostic criteria used to calculate AUD prevalence rate					
	Standard ECA and NCS criteria	NCS: narrow criteria	DSM-5 criteria	Narrow et al. (3), including clinical significance criteria	HD/ECA and HD/NCS criteria	HD/ECA and HD/NCS (adjusted to be ECA comparable) criteria
ECA (*N* = 11,092)	7.3 (6.7, 8.0)			8.9 (8.3, 9.5)	3.3 (2.8, 3.8)	3.3 (2.8, 3.8)
NCS (*N* = 7,599)	9.9 (8.9, 11.0)	7.0 (6.1, 7.9)	9.8 (8.9, 10.7)	6.5 (5.7, 7.3)	4.3 (3.7, 5.0)	3.6 (3.0, 4.2)

*Weighted and corrected for sampling design*.

*ECA, epidemiologic catchment area study; NCS, National Comorbidity Survey; HD, harmful dysfunction. See “Appendix” for details of diagnostic criteria*.

*Significant differences between groups are indicated by non-overlapping 95% confidence intervals*.

*^a^For the Standard ECA and NCS criteria sets, at least one symptom had to appear in the year prior to the interview; in the NCS, narrow criteria, DSM-5, Narrow et al. and HD diagnostic criteria sets, all symptoms had to appear in the year prior to the interview*.

Narrow et al. (3), in their unsuccessful attempt to reconcile the significant differences in the ECA and NCS 1-year prevalence estimates, arrived at ECA and NCS AUD rates of 8.9 and 6.5%, respectively. These adjusted rates are quite different from each other and in both cases significantly higher than the corresponding HD estimates. For comparative purposes (not reported in the table), the NESARC 1-year AUD rate was 8.5% (9), and the Dunedin study average 1-year rate for alcohol dependence alone for ages 18–32 was 12.7% (12).

As in the lifetime analysis, we recalculated the HD/NCS prevalence using HD/ECA comparable criteria to test for consistency across studies, and found the ECA and NCS rates converging to 3.3 and 3.6%, respectively. These tests address the first two challenges for the 1-year rates; they show dramatically reduced rates relative to other studies, and rates that converge across the two target studies when comparable HD-based criteria are used.

Here, too, for comparative purposes, we note the standard 1-year dependence prevalence rates of 5.0 and 8.3% for the ECA and NCS, respectively, taken from Regier et al. (2). Like the lifetime dependence rates, these 1-year dependence rates are still substantially above those yielded by the HD analysis.

An additional finding is that DSM-5 criteria, at least in our approximation, does not substantially improve on previous criteria with regard to implausibly high rates (Tables 2 and 3). The lifetime rate of 19.5% is a bit lower than the NCS lifetime rate but higher than the ECA rate, and the 1-year DSM-5 rate of 9.8% is the same as the NCS rate and significantly higher than the ECA rate. This is to be expected; the changes to the criteria in DSM-5 were designed to leave overall AUD prevalence about the same as before. For comparison purposes, Edwards et al. (11) found a lifetime DSM-5 AUD prevalence rate of 32%, and Agrawal et al. (65) and Mewton et al. (66) found 1-year DSM-5 prevalence rates of 10.8 and 9.7%, respectively.

Reducing prevalence rates can be easily accomplished in a number of ways, but the achievement is meaningless if the resulting classification is not valid (21). The results of validator tests for lifetime disorder are presented in Table 4, and consistently support the validity of the HD criteria. The HD cases possess significantly greater duration – by about 3–4 years on average – than NCS and DSM-5 cases. For each of three service use indicators – saw a mental health professional, attended AA, and went to an alcohol outpatient clinic – the rates for the HD group are double or more the rates of the other two groups, and significantly higher in every case. Regarding comorbid mood and anxiety disorders, these disorders as defined by the DSM are quite common so all the rates are high, but the rate of comorbidity for the HD group is still significantly higher than for either of the other two groups.

**Table 4:** **Means and percentages (95% confidence intervals) for validators of NCS lifetime ^a^alcohol use disorders, ages 18–54, *N* = 7,599**.

	NCS (*n* = 1,947)	HD/NCS (*n* = 550)	DSM-5 (*n* = 1,536)
Mean duration, years	8.3 (7.6, 9.0)	12.1 (11.1, 13.1)	9.1 (8.3, 9.9)
% See mental health professional about substance use, ever^b^	11.8 (10.4, 13.3)	27.1 (22.1, 32.2)	14.9 (12.9, 16.8)
% Attended AA or NA meetings, ever^c^	18.4 (15.7, 21.1)	44.6 (38.3, 50.9)	22.6 (19.1, 26.0)
% Went to drug or alcohol outpatient clinic, ever^d^	6.0 (4.6, 7.3)	15.8 (11.7, 20.0)	7.2 (5.5, 8.8)
% Have any NCS mood or anxiety disorder, lifetime^e^	47.7 (44.0, 51.4)	62.4 (57.4, 67.4)	50.7 (47.0, 54.4)
% Of transient teen users with NCS AUD having the given disorder, ages 15–54^f^	100 (*n* = 287)	10.3 (5.5, 12.6) (*n* = 29)	65.2 (57.7, 72.6) (*n* = 184)

*Weighted and corrected for sampling design*.

*NCS, National Comorbidity Survey; HD, harmful dysfunction*.

*Significant differences between groups are indicated by non-overlapping 95% confidence intervals*.

*^a^For each diagnostic criteria set, the symptoms could occur at any point in the respondent’s lifetime. They did not need to occur at the same time point*.

^b^Did you ever see a mental health specialist about your substance use? (By mental health specialists we mean psychiatrists, psychologists, or social workers.)

^c^Did you ever go to a self-help group like Alcoholics Anonymous or Narcotics Anonymous because of your substance use?

^d^Have you ever gone to a drug or alcohol outpatient clinic for professional help with your emotions or nerves or your use of alcohol or drugs?

*^e^% Any mood or anxiety disorder, lifetime, for the entire sample: 34.7 (32.6, 36.7)*.

*^f^Teen transient users defined as having lifetime NCS alcohol disorder, but not having 1-year NCS alcohol disorder, age of onset at 19 years old or younger, alcohol disorder duration <5 years; uses full sample, *N* = 8098, for analyses. By the definition, all of the teen transient users have lifetime NCS alcohol disorder, giving the 100% result for that cell in the above table. The other cells in that row are based on those 287 cases as the base rate in the denominators*.

For the 1-year disorders, the validators tell a similar story (Table 5). Duration is on average about two and a half years longer for HD AUDs than for the others. Service use for all three service use validators is significantly greater for HD criteria than for NCS criteria, and as compared to the DSM-5 group is significantly greater for two of the service use validators (saw a mental health professional, attended AA) and marginally greater for the third (attended an outpatient alcohol clinic). The validator rates for the NCS 1-year narrow approach are no different from the rates for the standard approach and do not changes the pattern of results of the comparisons to the HD model, so the broad versus narrow approach is not determining the results. Comorbidity is significantly higher among HD-diagnosed than among NCS-diagnosed individuals, and higher but not quite significantly so in relation to DSM-5-diagnosed individuals.

**Table 5:** **Means and percentages (95% confidence intervals) for validators of NCS 1-year ^a^alcohol use disorders, ages 18–54, *N* = 7,599**.

	NCS: broad criteria (*n* = 793)	NCS: narrow criteria (*n* = 539)	HD/NCS (*n* = 336)	DSM-5 (*n* = 762)
Mean duration, years	10.4 (9.3, 11.4)	10.5 (9.3, 11.7)	13.0 (11.9, 14.1)	10.7 (9.6, 11.8)
% See mental health professional about substance use, ever^b^	11.9 (9.1, 14.8)	11.6 (8.7, 14.4)	31.5 (24.6, 38.4)	19.1 (15.3, 22.8)
% Attended AA or NA meetings, ever^c^	20.5 (16.1, 24.9)	23.3 (18.0, 28.6)	52.2 (44.7, 59.7)	29.4 (24.3, 34.5)
% Went to drug or alcohol outpatient clinic, ever^d^	7.8 (5.3, 10.4)	8.5 (5.1, 12.0)	20.4 (14.3, 26.5)	11.5 (8.4, 14.6)
% Have any NCS mood or anxiety disorder, lifetime^e^	50.1 (44.4, 55.8)	49.9 (43.2, 56.6)	62.8 (56.0, 69.7)	52.4 (47.1, 57.6)

*Weighted and corrected for sampling design*.

*NCS, National Comorbidity Survey; HD, harmful dysfunction*.

*Significant differences between groups are indicated by non-overlapping 95% confidence intervals*.

*^a^For the NCS: broad criteria set, at least one symptom had to appear in the year prior to the interview; in the NCS: narrow, HD and DSM-5 diagnostic criteria sets, all symptoms had to appear in the year prior to the interview*.

^b^Did you ever see a mental health specialist about your substance use? (By mental health specialists we mean psychiatrists, psychologists, or social workers.)

^c^Did you ever go to a self-help group like Alcoholics Anonymous or Narcotics Anonymous because of your substance use?

^d^Have you ever gone to a drug or alcohol outpatient clinic for professional help with your emotions or nerves or your use of alcohol or drugs?

*^e^% Any mood or anxiety disorder, lifetime for the entire sample: 34.7 (32.6, 36.7)*.

Finally, the fourth challenge concerns the level of unmet need. We defined unmet need for services as anyone who satisfies diagnostic criteria for an AUD but answers “no” to all three questions regarding service use (saw a mental health professional, attended AA, went to an outpatient clinic). Obviously, there are many reasons for need for help with alcohol problems, some of which may qualify as medically necessary when the alcohol is threatening to exacerbate health problems. However, here we focus on unmet need based specifically on AUD diagnosis. The result for the NCS confirms the problem of unmanageable unmet need estimates, with 19.2% of the entire population having an unmet need for AUD services based on lifetime disorder, and 7.4% in a 1-year period (Table 6). The DSM-5 criteria reduce these estimates somewhat but still leave them enormously high, with 14.0% lifetime and 6.3% 1-year unmet need. Even the NCS 1-year narrowly defined group yields a rate of 5% of the entire population having unmet need in a given year. The HD criteria alter this unmanageably challenging landscape by reducing lifetime and 1-year AUD unmet need rates to 3.4 and 1.8%, respectively.

**Table 6:** **Unmet need: percentages (95% confidence intervals) of the general population having lifetime and 1-year alcohol use disorders but no use of services, using NCS, HD, and DSM-5 diagnostic criteria, ages 18–54, *N* = 7,599**.

	NCS	NCS: narrow criteria	HD/NCS	DSM-5
Percentage of the general population with lifetime alcohol use disorder but no service use^a^	19.2 (17.5, 20.9) (*n* = 1,475)		3.4 (2.7, 4.1) (*n* = 272)	14.0 (12.6, 15.5) (*n* = 1,088)
Percentage of the general population with 1-year alcohol use disorder but no service use^b^	7.4 (6.5, 8.4) (*n* = 577)	5.1 (4.4, 5.8) (*n* = 381)	1.8 (1.3, 2.2) (*n* = 144)	6.3 (5.5, 7.1) (*n* = 476)

*Weighted and corrected for sampling design. The number of cases of unmet need for each cell is given in square brackets*.

*NCS, National Comorbidity Survey; HD, harmful dysfunction*.

*“No service use”: respondent reported never having used any of the following three services: (1) seen a mental health professional about substance use, (2) gone to a self-help group like Alcoholics Anonymous or Narcotics Anonymous because of substance use, (3) gone to a drug or alcohol outpatient clinic for help with emotions, nerves, or use of alcohol or drugs*.

*^a^For each diagnostic criteria set, the symptoms could occur at any point in the respondent’s lifetime. They did not need to occur at the same time point. The baseline lifetime disorder prevalence estimates are NCS: 24.9; HD: 6.8; DSM-5: 19.5*.

*^b^For the NCS criteria set, at least one symptom had to appear in the year prior to the interview; in the NCS: narrow, HD/NCS, and DSM-5 diagnostic criteria sets, all symptoms had to appear in the year prior to the interview. The baseline 1-year disorder prevalence rates are NCS: 9.9; NCS: narrow: 7.0; HD: 4.3; DSM-5: 9.8*.

### Specificity analysis

As noted earlier, in *post hoc* analyses we performed rough tests to examine diagnostic specificity and sensitivity. For testing specificity, the challenge was to identify a criterion group of respondents who exhibit drinking behaviors that might qualify for AUD diagnosis, but who likely are not genuinely disordered. The group we identified consists of respondents who participated in sufficiently heavy drinking during their teenage years to be classified by NCS criteria as having an AUD, but who were transient in their heavy usage and had remitted during young adulthood and no longer qualified for AUD. There has been much discussion recently of the possibility that such transient “teen bingers” are being misclassified as having AUD (67–70). We assumed that many members of this group are drinking as part of youthful social relationships and – if they quickly and enduringly gave up such behaviors as they matured – are most likely not suffering from a physical or psychological dysfunction. We operationalized this criterion group as any respondent who met lifetime NCS alcohol disorder criteria, had an age of onset of 19 years old or younger, had alcohol disorder duration <5 years, and remitted and did not have 1-year NCS alcohol disorder at the time of the NCS interview. We predicted that more valid HD diagnostic criteria should eliminate disproportionately many of this group from their classification as disordered by the NCS.

The result of the specificity test for HD AUD validity, using transient teen users classified by the NCS as disordered as the criterion group, is presented in Table 4. By definition, 100% of these individuals qualify for NCS lifetime disorder, yet given their overall history, it is plausible that most were not disordered. We found that DSM-5 criteria still classify 65% of these transient teen users as disordered. In contrast, the HD analysis essentially depathologizes this group, classifying only 10% of them as disordered. Notably, this is a disproportionate reduction; overall, the HD criteria classify about 25% of the NCS disorders as HD disorders.

### Sensitivity analysis

Regarding sensitivity, a challenge facing the HD analysis is the fear of false negative diagnoses due to reduction in cases. To examine this issue, we used service use indicators to test for sensitivity of 1-year diagnosis. The idea was to see whether the HD analysis missed a large number of individuals who sought services. The test is approximate because the service use questions are lifetime rather than 1-year and they do not specify whether the individual sought help for alcohol or other substances. Moreover, many individuals seeking help with alcohol issues, or referred for such help, may not be disordered. However, if the rate of individuals seeking help but not HD disordered was extremely high, this might be a red flag that there are problematic levels of false negatives.

We calculated the absolute number of individuals (unweighted) with 1-year AUD who sought three kinds of measured service use, comparing rates among those diagnosed with standard NCS AUD versus HD/NCS AUD. For standard NCS 1-year disorder, the numbers who sought services from the three service venues of mental health professionals, AA or NA, and outpatient alcohol or drug clinics were 94, 163, and 62, respectively; and for HD/NCS the numbers were 106, 175, and 69, respectively. In other words, despite the fact that HD/NCS classified only 42% as many individuals as disordered as did the standard NCS criteria, HD/NCS AUD still included a larger number of individuals who sought services in each of the three service categories. By this test, it appears that despite the HD approach’s lowering of prevalence, false negatives may be minimal.

### Item-level analyses

Our primary analysis was undertaken at the full syndromal level. Further studies will be necessary to explore the impact of each individual symptom criterion on HD diagnosis and validity. However, in *post hoc* analyses we calculated item-level prevalences in the NCS HD sample to check whether any particular criteria had a major role in the results.

For the four dysfunction indicators, the percentages of HD disorders having that dysfunction ranged from 38 to 58%, and the average number of dysfunctions was 1.9. Thus, in general if a dysfunction was present then more than one dysfunction was present. Regarding the nine harm items, in terms of percentages of HD cases manifesting each harm, the average was 40%, but there was a lower outlier (alcohol use often kept you from working, going to school, taking care of children, 4%) and two higher outliers (problems with your family, friends, at work, at school or with the police, 70%; under the effects of alcohol in situations that increased your chances of getting hurt, like driving a car, 77%). The average number of harms for HD cases was 3.6, so the two upper-end outliers were not responsible for our results; without them, there was still an average of more than two harms per HD case. Given the redundancy of both dysfunction and harm in many cases, membership in the HD AUD category appears to be a relativity robust feature not dependent on a particular item.

### Remission rates of standard versus HD AUD

An issue that has become salient in recent discussions of AUD is whether individuals spontaneously remit from what is supposed to be a disorder lacking control. Although in fact lack of control during an episode of disorder is in principle conceptually distinct from whether one can or does remit from the disorder, critics of the out-of-control model have tended to link the two, suggesting that remission conflicts with the notion of being out-of-control and that the observed pattern of remission conflicts with the out-of-control model (54). Consequently, high rates of AUD remission reported in epidemiological surveys have fueled arguments that perhaps the entire conceptualization of AUD as a disorder is mistaken.

To examine the remission issue, we performed a *post hoc* analysis in which we compared NCS remission rates using standard criteria to NCS remission rates using the HD criteria. As the indicator of remission, we used the percentage of lifetime cases that were not 1-year cases. For the NCS study, the standard 1-year and lifetime rates were 9.9 and 24.9%, respectively, yielding a remission rate of 60%. For the HD analysis, the 1-year and lifetime NCS disorder rates were 4.3 and 6.8%, respectively, yielding a substantially lower remission rate of 37%. This result suggests the possibility that as criteria are made more valid for picking out AUD in the sense of disorder, remission rates may tend to drop substantially.

### HD analysis versus DSM dependence

As noted in “Materials and Methods,” one might object to the above analyses that HD AUD is basically like traditional dependence, and the improvements in convergence and validity that we found for the HD criteria are explained by the fact that we compared the HD criteria to the larger and less valid category of DSM combined dependence/abuse. We thus repeated our analyses of convergence of prevalence rates, validator rates, specificity and sensitivity tests, and unmet need rates, but this time comparing HD criteria to criteria for DSM dependence as defined in the ECA and NCS (Tables A1 and A2 in Appendix). These analyses do not include a comparison to DSM-5 criteria, because DSM-5 eliminated the dependence/abuse distinction.

First, was our striking finding of convergent prevalence rates across studies virtually guaranteed by the HD analysis’s similarity to DSM dependence? The answer is that no such piggybacking on dependence can explain our convergence results. The results indicate, first, that DSM dependence is itself highly divergent across the ECA and NCS studies. Lifetime prevalence of DSM dependence in the ECA is 8.8% (8.1, 9.5), but in the NCS it is 14.9% (13.6, 16.3). Similarly, 1-year prevalence of DSM dependence in the ECA is 4.2% (3.8, 4.7), whereas in the NCS it is 7.4% (6.5, 8.4); with Regier et al.’s corrections to increase comparability, the 1-year prevalences are 4.6 and 8.3%, respectively. In both cases, the ECA/NCS differences are not only significant but substantial, in contrast to the convergence of HD prevalences.

When it comes to convergence of prevalence rates across studies, DSM dependence actually performs worse than combined dependence/abuse. Using the percentage change in prevalence from the ECA to the NCS as a measure of divergence (the larger the percentage change, the greater the divergence), the ECA-to-NCS percentage changes in both lifetime and 1-year prevalence is greater for dependence (lifetime, 69% change; 1-year, 76% change both without and with corrections) than for combined dependence/abuse (lifetime, 62% change; 1-year, 37% change). Therefore, no purported resemblance to dependence can explain the lower changes in HD prevalence rates (without corrections, lifetime, 13% change; 1-year, 30% change; with corrections to increase comparability, lifetime, 8% change; 1-year, 9% change).

We also duplicated our earlier validity analysis using the same five primary validators, but applied to NCS dependence rather than combined dependence/abuse. For four validators (duration, ever saw a mental health professional for substance use, ever attended AA or NA meetings, ever went to an outpatient drug or alcohol clinic), the percentage with HD disorder who had that validator was significantly and substantially higher than the percentage of those with NCS dependence disorder who had the validator (Table 7). Generally speaking, the average duration of HD disorder was about two and a half years longer than for DSM dependence (e.g., average lifetime duration was 9.7 years for DSM dependence, and 12.1 years for HD disorder), and the rates of service use for HD disorder were consistently about twice as high as for DSM dependence (e.g., percentages of 1-year DSM dependence cases using mental health professional and AA/NA services were 14.7 and 25.7%, respectively, whereas corresponding percentages for 1-year HD were 31.5 and 52.2%, respectively). For the one remaining validator (comorbid mood or anxiety disorder), HD disorder was higher on the validator but not significantly so (Table 7). These results support the greater validity of HD disorder over NCS dependence.

**Table 7:** **Means and percentages (95% confidence intervals) for validators of NCS lifetime and 1-year ^a^alcohol dependence disorders versus HD AUD, ages 18–54, *N* = 7,599**.

	NCS dependence: lifetime (*n* = 1,182)	NCS dependence: 1-year (*n* = 597)	HD lifetime (*n* = 550)	HD 1-year (*n* = 336)
Mean duration, years	9.7 (9.0, 10.5)	10.5 (9.3, 11.7)	12.1 (11.1, 13.1)	13.0 (11.9, 14.1)
% See mental health professional about substance use, ever^b^	16.9 (14.5, 19.4)	14.7 (11.2, 18.1)	27.1 (22.1, 32.2)	31.5 (24.6, 38.4)
% Attended AA or NA meetings, ever^c^	27.6 (23.6, 31.6)	25.7 (19.8, 31.7)	44.6 (38.3, 50.9)	52.2 (44.7, 59.7)
% Went to drug or alcohol outpatient clinic, ever^d^	8.8 (6.8, 10.8)	9.7 (6.6, 12.8)	15.8 (11.7, 20.0)	20.4 (14.3, 26.5)
% Have any NCS mood or anxiety disorder, lifetime^e^	53.1 (48.8, 57.4)	53.9 (48.0, 59.8)	62.4 (57.4, 67.4)	62.8 (56.0, 69.7)
% Of transient teen users with NCS AUD having the given disorder, ages 15–54	100 (*n* = 88)		17.8 (7.7, 27.9) (*n* = 19)	
% Of general population with given alcohol disorder but no service use	10.0 (8.9, 11.2) (*n* = 777)	5.1 (4.3, 5.9) (*n* = 399)	3.4 (2.7, 4.1) (*n* = 272)	1.8 (1.3, 2.2) (*n* = 144)

*Weighted and corrected for sampling design*.

*^a^For the NCS at least one symptom had to appear in the year prior to the interview*.

^b^Did you ever see a mental health specialist about your substance use? (By mental health specialists we mean psychiatrists, psychologists, or social workers.)

^c^Did you ever go to a self-help group like Alcoholics Anonymous or Narcotics Anonymous because of your substance use?

^d^Have you ever gone to a drug or alcohol outpatient clinic for professional help with your emotions or nerves or your use of alcohol or drugs?

*^e^% Any mood or anxiety disorder, lifetime for the entire sample: 34.7 (32.6, 36.7)*.

*NCS alcohol dependence lifetime prevalence: 14.9% (13.6, 16.3)*.

*HD alcohol disorder lifetime prevalence: 6.8% (5.9, 7.7)*.

*NCS alcohol dependence 1-year prevalence: 7.4% (6.5, 8.4)*.

*HD alcohol disorder 1-year prevalence: 4.3% (3.7, 5.0)*.

Is NCS dependence more valid on our indicators than NCS combined dependence/abuse? Comparing Table 7 to Tables 4 and 5, the increment in validity in moving from combined dependence/abuse to dependence, as indicated by increasing percentages of validators, is surprisingly modest. For 1-year disorder, there is a trend for dependence to be slightly higher on validator percentages, but the increases do not reach significance on any of the validators. Of particular interest is that the reported duration of dependence cases and combined dependence/abuse cases is virtually identical. Looking at lifetime validators, dependence is significantly higher than combined dependence/abuse on two out of the five validators (seeing a professional, attending AA or NA), and for the other three validators there is not a significant difference. A limitation is that these NCS analyses used DSM-III-R criteria, but these are quite similar in major respects to DSM-IV criteria (three out of nine symptoms, many having similar wording to that used in DSM-IV). Clearly, the validity results for the HD criteria cannot be explained by any parasitic relationship to dependence, which does only very modestly better than combined dependence/abuse.

In terms of unmet need (Table 7), dependence rates are lower than combined dependence/abuse, but still very high. The HD analysis reduces both lifetime and 1-year NCS dependence rates of unmet need by about two-thirds. For example, in a given year, instead of 5.1% of the entire adult population having unmet need for treatment according to the dependence criteria, only 1.8% of the population has unmet need for treatment by the HD analysis.

For the HD versus dependence comparison, we duplicated the specificity and sensitivity analyses we did for combined dependence/abuse. For specificity, of the individuals who reported transient adolescent drinking and who qualified for NCS dependence, only about 18% of them also qualified for HD disorder, so the HD criteria are still making a major difference (Table 7). This is a highly disproportionate reduction; overall, about 47% of those who qualified for NCS dependence also qualified for HD disorder. Thus, if one suspects that transient adolescent use of alcohol is being overdiagnosed, then the HD analysis appears to offer a more effective corrective than dependence.

The sensitivity analysis again used 1-year data, and we used the two most frequent service use indicators – ever seeing a professional about substance use and ever attending AA or NA meetings (again, these items were limited by not being specific to alcohol). For both validators, despite the fact that 1-year HD disorders occurred only 56% as frequently as NCS dependence, the absolute number of diagnosed individuals who reported service use was higher for HD disorder than for NCS dependence. For 1-year NCS dependence, 88 individuals reported seeing a mental health professional and 153 reported attending AA or NA; the comparable figures for 1-year HD disorder were 106 seeking professional help and 175 attending AA or NA. The HD criteria thus managed to pick out the service use seekers from among a much larger pool of NCS-dependent individuals, and even identified a considerable number of help-seeking individuals the NCS dependence criteria had missed.

The remission rate of NCS DSM dependence – that is, the percentage of those with lifetime dependence who did not have 1-year dependence – was 50%. This was lower than the remission rate for combined dependence/abuse (60%) but higher than the HD remission rate (37%).

These analyses disconfirm the idea that the success of the HD analysis in yielding prevalence convergence across studies and increasing validator levels is due to its being similar to dependence and our having used the broader dependence/abuse category for comparisons. Indeed, within the constraints of these analyses, these results cast doubt on the common belief that DSM dependence is a much more valid category than combined dependence/abuse. In any event, the incremental value of the HD approach over dependence is strongly affirmed.

## Discussion

The distinction between problem drinking and AUD is important both conceptually and pragmatically. Mixing together those who choose to drink heavily or suffer adverse effects of doing so with those who have a mental disorder of drinking motivation – thus yielding “false-positive” AUD diagnoses – undermines attempts to establish brain correlates of disorder, identify etiological pathways and risk factors for disorder, offer patients appropriate prognosis and informed consent for treatment, and test treatments aimed at ameliorating disorder (71). The standard view of the dysfunction that exists in AUD since about the mid-twentieth century, and the view underlying the DSM’s approach to diagnosis of AUDs as well as the present analysis, is that some people who drink to excess suffer from a motivational dysfunction that leads to loss of normal-range deliberative control over drinking (57). The attempt to improve validity of AUD diagnosis in the present study took place within the framework of the loss-of-control model.

Alcohol use is an area of social ambivalence that warrants caution among nosologists lest they become agents of social control by labeling harmful or disapproved behavior as disordered when there is no evidence of dysfunction. Yet DSM AUD diagnostic criteria seem to inhabit an alternative conceptual universe in which problems common among the non-addicted, such as arguing with family members over alcohol use, strong preference for alcohol-related activities, and driving under the influence of alcohol are taken as *prima facie* evidence of a psychiatric disorder rather than simply harmful effects of drinking. Without valid criteria distinguishing AUD from non-disordered problems, in the long run it will be more difficult for research to unlock AUD etiology and to identify effective treatments, and in the short run treatment selection will be muddied by diagnostic mixing of very different conditions.

The present study attempted to approach the criteria with a strict focus on conceptual validity, especially in requiring indicators of dysfunction. Our central hypothesis was that the out-of-control model had not been given a fair test because of confusion in diagnostic criteria between motivational dysfunctions on the one hand and harmful effects of drinking and strong preferences for drinking on the other hand. We provided initial data on the validity of a possible HD translation of the loss-of-control model.

### Major novel findings

Other researchers have noted the potential usefulness of conceptualizing AUDs in terms of the HD analysis (72, 73), and some efforts to revise criteria have been along lines consistent with implicit HD thinking (74–76). However, this is the first study explicitly and systematically to formulate criteria for AUD based strictly on HD considerations, apply them to major epidemiological data sets, and evaluate the outcome in a systematic way. The evaluation offers the first three-way validator analysis comparing the validity of the approaches of traditional DSMs, the new DSM-5, and the HD analysis to defining AUD, using standard validators such as duration, service use, and comorbidity.

The results suggest that, in terms of the validators we were able to deploy from the NCS data set, the DSM-5 criteria generally yielded non-significant trends toward elevated validator levels relative to the NCS’s DSM-III-R criteria while modestly reducing prevalence. Thus, the DSM-5 changes seem not to represent any great progress in terms of validity. In contrast, the HD analysis significantly and substantially increased validator rates over both DSM-III-R and DSM-5 while also significantly and substantially lowering prevalence rates relative to DSM-III-R and DSM-5 as well as yielding convergent rates across studies. The results suggest that continued exploration of a somewhat revised out-of-control model of addictive pathology is warranted. Substantively, the results indicate that a stricter approach to AUD diagnosis could yield a lifetime prevalence more in the 6% range than the 30% range.

This paper also presents the first attempt subsequent to Regier et al.’s (2) and Narrow et al.’s (3) classic papers to resolve the two-decade-old puzzle of divergent ECA and NCS prevalence rates. According to our findings, there is an underlying convergence once a more valid and narrower HD-derived definition of AUD is applied to the data. This finding of convergent prevalence rates across studies emerged despite somewhat divergent criteria, as we had hypothesized. These findings implicitly address broader doubts about the viability of community studies of psychiatric disorder. Of course, convergence can have many other explanations, and further research will be necessary to confirm that increased validity is responsible for the present convergence. However, the validator results reported here tend to support this explanation.

This is also the first successful attempt we know of to formulate and test an explanation of the much-discussed puzzle of high transient adolescent rates of AUD. The high rates of apparently spontaneously remitting adolescent AUD have been prominently featured in recent arguments that AUD is not really a disorder at all but a matter of normal-range choice (4, 5). Our analysis indicates that the vast majority of these cases do not satisfy HD requirements for disorder. This suggests that it might be misguided to rely on these cases to argue that there is no true AUD, because in fact the identification of these cases as AUDs in epidemiological surveys is likely due to invalid definitions of AUD yielding large numbers of false-positive cases during adolescence.

Hasin’s (77) statement that “we still lack the ability to differentiate between young individuals in the general population who evidence the criteria and remit and those who go on to develop chronic, debilitating alcohol or other drug disorders” (p. 703) is no longer entirely true in light of the present results. Of those young individuals who satisfied NCS AUD criteria but remitted within 5 years and did not have a current disorder at the time of the NCS interview, 90% of them were eliminated from the disorder category by applying the HD analysis criteria, higher than the overall 72% reduction of prevalence. In contrast, of those young people who had at least a 10-year duration and had current 1-year disorder at the time of the NCS interview, only 42% were eliminated as non-disordered. Thus, HD criteria do offer some predictive power when it comes to likely transience of adolescent-onset cases.

This paper’s calculations of unmet need for treatment offer an estimate that, while still challengingly high at about 4.5 million U.S. adults with unmet need for AUD treatment per year, greatly diverges from standard unmet need rates of about 17 million individuals per year. Further research is needed to address whether these revised estimates represent an improved differentiation of those with unmet need for treatment of harmful compulsive use from those experiencing other alcohol-related issues, which they might or might not want treated. In evaluating these unmet need results, one must consider the remarkable statistics on unmet versus felt need from the 2012 National Survey on Drug Use and Health: “Among the 16.8 million persons aged 12 or older who needed but did not receive specialty treatment for an alcohol use problem in 2012, 665,000 persons (4.0%) felt they needed treatment for their alcohol use problem…. Of the 665,000, …, 490,000 did not make an effort to get treatment, and 174,000 made an effort but were unable to get treatment” (78). Thus, of those qualifying for AUD status under current criteria but who did not receive treatment, about 1% actually felt they needed treatment and made some effort to seek treatment but were unsuccessful. While there are many psychological, social, and institutional obstacles to seeking treatment for AUD, there is a *prima facie* implausibility to these extraordinary rates of unfelt need among those purportedly needing treatment for compulsive alcohol use. Our analysis suggests a possible resolution to this puzzle; a very large number of these individuals do not have a true AUD with deliberational or motivational dysfunction and the resulting harm, and they judge, perhaps correctly, that treatment for AUD is unwarranted.

A surprising finding of this study was that all of the major conclusions about validity arrived at from the above comparisons of HD disorder to combined dependence/abuse held just as strongly when the HD analysis was compared to DSM dependence. The present analyses suggest that DSM dependence may not be as conceptually valid as has sometimes been claimed.

### Limitations

This study had all the limitations of the original cross-sectional ECA and NCS analyses, such as respondents’ faulty memories and limitations of lay interviewers. Longitudinal analysis would no doubt increase HD estimates. We analyzed only psychiatric disorders of deliberative control over alcohol drinking, but there are many other medical conditions related to alcohol use warranting treatment so AUD “should therefore not monopolize medical and social concern” [(37), p. 1060]. Secondary analysis was limited by the need to use questions as originally worded, rather than wording that would be optimal for the HD approach (e.g., with more contextual exclusions to eliminate false-positives).

A conceptual limitation is that the HD analysis’s concepts of “dysfunction” and “harm” that we used to conceptualize AUD, like other concepts currently used to conceptualize AUDs such as “compulsive,” “dependent,” or “out-of-control,” are fuzzy concepts. They thus required difficult judgments to operationalize, which we reached by discussion and consensus. So, there remains room for dispute about HD criteria. However, our judgments resonated with concerns about validity of diagnostic criteria expressed by others (72, 74, 79–83). In aiming for plausible prevalence estimates, clearly one can decide within the range of one’s guiding concepts to err on the side of avoiding false negatives and establishing relatively high AUD prevalence versus erring on the side of avoiding false-positives and establishing relatively low prevalence of alcoholism. However, validity considerations place limits on such flexibility if one is attempting to validly identify mental disorder. Future studies should explore alternative choices of HD-inspired criteria that can be justified theoretically.

Our translation of DSM-5 criteria into NCS questions also involved subjective judgment. Alternative translations with correspondingly altered results are possible. However, *post hoc* shadow analyses suggested that such alternative translations would not alter the primary findings of the analysis with regard to DSM-5 validators or comparisons of DSM-5 to other approaches.

We restricted our analysis to the ECA and NCS, the two data sets that were the target of Regier et al.’s (2) and Narrow et al.’s (3) early reanalyses. This provided a useful comparison point because they tried a variety of other strategies to increase validity and cross-study comparability, all of which failed to achieve the desired result. There are two other major epidemiological data sets available for public use, the National Comorbidity Survey Replication (NCS-R) (84) and the NESARC, and it might be asked why these were not included as well. The NCS-R reported substantially lower rates of dependence-only prevalence (5.4%) than other studies. However, it has emerged that the NCS-R used flawed methodology that generated artificially low dependence prevalence rates. Specifically, respondents were not asked dependence symptom questions unless they first responded positively to an abuse question, yet the abuse and dependence questions (taken from DSM-IV) do not overlap, so this procedure eliminated many dependence cases (9, 85–87). Kessler, the lead NCS-R researcher, admitted that this was an error and changed the procedure in later studies (88). These flaws render the NCS-R inappropriate for AUD prevalence studies, and its low reported dependence rate is meaningless. Regarding the NESARC, we report the NESARC’s AUD prevalences for comparison purposes but we did not reanalyze the NESARC in detail for this report. This is because that survey provides a very different array of validators and analysis opportunities that are not available in the NCS and ECA. A detailed NESARC HD analysis that extends the present framework is a priority that requires a separate report and is currently underway.

A further limitation is that our lowered prevalence estimates are limited to AUD and ignore other alcohol-related pathologies and problems. Thus, for example, although the HD approach lowers AUD prevalence, the prevalence of other psychiatric disorders induced by heavy use of alcohol (89) and the possible coexistence of multiple substance abuse in alcoholics (90) remain unaccounted for in our prevalence rates.

Finally, it should be emphasized that diagnosis is not the same as treatment need (91), and it is not the same as risk of disorder despite often being confused with risk (92, 93). Some disorders are mild and need not be aggressively treated, whereas many alcohol-related problems, even if not AUDs, create risk or actual harm that demands intervention.

### Implications regarding DSM-5

One of the primary findings of our analysis is that DSM-5’s single AUD category is not much of a change from DSM-IV’s dependence and abuse combined when it comes to prevalence and validator results. The prevalence result was expected because DSM-5 AUD criteria were designed to preserve overall DSM-IV dependence plus abuse prevalence. However, the lack of validator improvement is more concerning and may reflect an underlying validity problem. Based on “the lack of data to support an intermediate state between drug use and drug dependence” [(94), p. 867], and despite acknowledgment that “the dependence process and its consequences do seem conceptually distinct” [(77), p. 703], the DSM-5 criteria make no provision to differentiate AUD from problem drinking, and the dependence dimension essentially swallows up symptomatic problem drinking. In giving up the distinction between disorder and problem drinking and giving up the search for valid differentiating criteria, DSM-5 likely violates DSM-5’s definition of mental disorder by classifying some alcohol problems as disorders even when there is no evidence of underlying dysfunction (e.g., when there are two abuse symptoms). The provisional positive evaluation of the HD analysis in this study suggests that the DSM-5 changes may have been premature and may obscure a potential alternative approach that has many advantages. It thus appears that the HD approach is worth further empirical exploration in seeking improved validity of AUD diagnostic criteria.

## Conclusion

It remains an open question whether the loss-of-control model is a satisfactory model of a genuine AUD dysfunction. However, the results of this study suggest that a problem in testing the loss-of-control model has been its questionable translation into diagnostic criteria. Conceptually driven revisions to diagnostic criteria that particularly attend to dysfunction indicators could lead to cleaner tests of the model’s validity. This paper’s results argue for a renewed effort to construct such diagnostic criteria sets that more effectively distinguish AUDs from other alcohol-related problems and thus might reduce false-positive AUD diagnoses that lead to confusion in the scientific and clinical literature.

## Author Contributions

Both Jerome C. Wakefield and Mark F. Schmitz contributed substantially to the conceptualization, data analysis, first-drafting, and revising of this paper.

## Conflict of Interest Statement

The authors declare that the research was conducted in the absence of any commercial or financial relationships that could be construed as a potential conflict of interest.

## Appendix

**Table A1:** **DSM-III diagnostic criteria for alcohol use disorders and their translation using epidemiologic catchment area study (ECA) questions**.

DSM-III diagnostic criteria for alcohol use disorders	Corresponding ECA questions used for the given DSM-III criteria
**DIAGNOSTIC CRITERIA FOR ALCOHOL ABUSE (A, B, AND C)**	
A. Pattern of pathological alcohol use: (any one of) need for daily use of alcohol for adequate functioning; inability to cut down or stop drinking; repeated efforts to control or reduce excess drinking by “going on the wagon“ (periods of temporary abstinence) or restricting drinking to certain times of the day; binges (remaining intoxicated throughout the day for at least 2 days); occasional consumption of a fifth of spirits (or its equivalent in wine or beer); amnesic periods for events occurring while intoxicated (blackouts); continuation of drinking despite a serious physical disorder that the individual knows is exacerbated by alcohol use; drinking of non-beverage alcohol	Answer “yes” to any one or more of the following
	Have you ever wanted to stop drinking but couldn’t?
	Some people promise themselves not to drink before 5 o’clock or never to drink alone, in order to control their drinking. Have you ever done anything like that?
	Have you ever drunk as much as a fifth of liquor in 1 day, that would be about 20 drinks, or three bottles of wine or as much as three six-packs of beer in 1 day?
	Have you ever had blackouts while drinking, that is, where you drank enough so that you couldn’t remember the next day what you had said or done?
	Have you ever continued to drink when you knew you had a serious physical illness that might be made worse by drinking?
	Has there ever been a period in your life when you could not do your ordinary daily work well unless you had had something to drink?
	How many times have you gone on benders that lasted at least a couple of days? (two or more counted as symptom)
B. Impairment in social or occupational functioning due to alcohol use: e.g., (any one of) violence while intoxicated, absence from work, loss of job, legal difficulties (e.g., arrest for intoxicated behavior, traffic accidents while intoxicated), arguments, or difficulties with family or friends because of excessive alcohol use	Answer “yes” to any one or more of the following
	Has your family ever objected because you were drinking too much?
	Have friends, your doctor, your clergyman, or any other professional ever said you were drinking too much for your own good?
	Have you ever had job (or school) troubles because of drinking – like missing too much work or drinking on the job (or at school)?
	Did you ever lose a job (or get kicked out of school) on account of drinking?
	Have you ever gotten into trouble driving because of drinking – like having an accident or being arrested for drunk driving?
	Have you ever been arrested or held at the police station because of drinking or for disturbing the peace while drinking?
	Have you ever gotten into physical fights while drinking?
C. Duration of disturbance of at least 1 month	(Not measured)
**DIAGNOSTIC CRITERIA FOR ALCOHOL DEPENDENCE (A AND B)**	
A. Either a pattern of pathological alcohol use or impairment in social or occupational functioning due to alcohol use (i.e., any one symptom of either pathological use or impairment; see above Alcohol Abuse criteria for pathological use and for impairment in social or occupational functioning)	Answer “yes” to any one or more of the questions above for either pathological use or impairment in social or occupational functioning (see ECA questions used for alcohol abuse)
B. Either tolerance or withdrawal	Answer “yes” to any one or more of the following
Tolerance: need for markedly increased amounts of alcohol to achieve the desired effect, or markedly diminished effect with regular use of the same amount	Has there ever been a period of 2 weeks when every day you were drinking seven or more beers, seven or more drinks, or seven or more glasses of wine?
Withdrawal: development of alcohol withdrawal (e.g., morning “shakes” and malaise relieved by drinking) after cessation of or reduction in drinking	Did you ever need a drink just after you had gotten up (that is, before breakfast)?
	Have you ever had “the shakes” after stopping or cutting down on drinking (for example, your hands shake so that your coffee cup rattles in the saucer or you have trouble lighting a cigarette)?

**Table A2:** **DSM-III-R diagnostic criteria for alcohol use disorders and their translation using National Comorbidity Survey (NCS) questions**.

DSM-III-R diagnostic criteria for alcohol use disorders	Corresponding NCS questions used for the given DSM-III-R criteria
**DIAGNOSTIC CRITERIA FOR PSYCHOACTIVE ALCOHOL ABUSE (A, B, AND C)**	
A. A maladaptive pattern of psychoactive substance use indicated by at least one of the following	Answer “yes” to any one or more of the following
(1) Continued use despite knowledge of having a persistent or recurrent social, occupational, psychological, or physical problem that is caused or exacerbated by use of the psychoactive substance	Did alcohol ever cause you problems with your family, friends, at work, at school or with the police?
(2) Recurrent use in situations in which use is physically hazardous (e.g., driving while intoxicated)	Did your use of alcohol ever cause you to be expelled from school, or to be demoted or fired from work?
	Have you often been under the effects of alcohol or feeling its after-effects in a situation which increased your chances of getting hurt – like when driving a car or boat, using knives or guns or machinery, crossing against traffic, climbing or swimming?
	Did you continue to use alcohol after it caused an accident (when you injured yourself while under the influence of alcohol – like had a bad fall or cut yourself badly, been hurt in a traffic accident, or anything like that)?
	Have you ever had any health problems as a result of using alcohol – such as liver disease, stomach disease, pancreatitis, feet tingling, numbness, memory problems, an accidental overdose, a persistent cough, a seizure of fit, hepatitis, or abscesses?
	Have you ever had any emotional or psychological problems from using alcohol – such as feeling uninterested in things, feeling depressed, suspicious of people, paranoid, or having strange ideas?
B. Some symptoms of the disturbance have persisted for at least 1 month, or have occurred repeatedly over a longer period of time	Built into the question responses in the NCS, emphasizing the word “often” in the symptom question, or coding symptom duration lasting at least 1-year within the diagnostic algorithm.
C. Never met the criteria for psychoactive substance dependence for this substance	Built into the hierarchy for NCS diagnosis; abuse can be diagnosed only if dependence is not diagnosed
**DIAGNOSTIC CRITERIA FOR PSYCHOACTIVE ALCOHOL DEPENDENCE (A AND B)**	
A. At least three of the following	At least three of the following (to satisfy a given DSM-III-R numbered criterion, answer “yes” to any one or more of the corresponding NCS questions)
(1) Substance often taken in larger amounts or over a longer period than the person intended	Did you often use larger amounts of alcohol than you intended to when you began, or did you use it for a longer period of time than you intended to?
	Did you often start using alcohol and find it difficult to stop before you became completely intoxicated or high?
(2) Persistent desire or one or more unsuccessful efforts to cut down or control substance use	Have you ever felt such a strong desire or urge to use alcohol that you could not resist it or could not think of anything else?
	Did your use of alcohol ever become so regular that you would not change when, or how much you took it, no matter what you were doing or where you were?
	Have you ever wanted or tried to stop or cut down on alcohol but found you could not?
(3) A great deal of time spent in activities necessary to get the substance (e.g., theft), taking the substance (e.g., chain smoking), or recovering from its effects	Did you ever have a period of a month or more when you spent a great deal of time using alcohol, getting it, or getting over its effects?
(4) Frequent intoxication or withdrawal symptoms when expected to fulfill major role obligations at work, school, or home (e.g., does not go to work because hung over, goes to school or work “high,” intoxicated while taking care of his or her children), or when substance use is physically hazardous (e.g., drives while intoxicated)	Have you often been under the effects of alcohol or suffering its after-effects while at work or school or taking care of children?
	Has your use of alcohol often kept you from working, going to school, or taking care of children?
	Have you often been under the effects of alcohol or feeling its after-effects in a situation which increased your chances of getting hurt – like when driving a car or boat, using knives or guns or machinery, crossing against traffic, climbing or swimming?
(5) Important social, occupational, or recreational activities given up or reduced because of substance use	Have you ever given up or greatly reduced important activities in order to get, or to use alcohol – activities like sports, work, or seeing family and friends?
(6) Continued substance use despite knowledge of having a persistent or recurrent social, psychological, or physical problem that is caused or exacerbated by the use of the substance (e.g., keeps using heroin despite family arguments about it, cocaine-induced depression, or having an ulcer made worse by drinking)	Did alcohol ever cause you problems with your family, friends, at work, at school, or with the police?
	Did your use of alcohol ever cause you to be expelled from school, or to be demoted or fired from work?
	Did you continue to use alcohol after it caused an accident (when you injured yourself while under the influence of alcohol – like had a bad fall or cut yourself badly, been hurt in a traffic accident, or anything like that)?
	Have you ever had any health problems as a result of using alcohol – such as liver disease, stomach disease, pancreatitis, feet tingling, numbness, memory problems, an accidental overdose, a persistent cough, a seizure of fit, hepatitis, or abscesses?
	Have you ever had any emotional or psychological problems from using alcohol – such as feeling uninterested in things, feeling depressed, suspicious of people, paranoid, or having strange ideas?
(7) Marked tolerance: need for markedly increased amounts of the substance (i.e., at least a 50% increase) in order to achieve intoxication or desired effect, or markedly diminished effect with continued use of the same amount	Did you ever find that you had to use more alcohol than usual to get the same effect or that the same amount had less effect on you than before?
(8) Characteristic symptoms of withdrawal	Did stopping or cutting down on alcohol ever make you sick or cause you problems like those listed on page 17?
(9) Substance often taken to relieve or avoid withdrawal symptoms	Did you ever use alcohol to make these withdrawal symptoms go away or to keep from having them?
B. Some symptoms of the disturbance have persisted for at least 1 month, or have occurred repeatedly over a longer period of time	In the NCS at least two of the above nine symptoms had to occur often or persist for at least 1-year

**Table A3:** **Harmful dysfunction (HD) diagnostic categories of harm and dysfunction and how they were translated using epidemiologic catchment area study (ECA) questions**.

HD/ECA	ECA alcohol question
Harm	Has your family ever objected because you were drinking too much?
Harm	Have friends, your doctor, your clergyman, or any other professional ever said you were drinking too much for your own good?
Harm	Have you ever had job (or school) troubles because of drinking – like missing too much work or drinking on the job (or at school)?
Harm	Did you ever lose a job (or get kicked out of school) on account of drinking?
Harm	Have you ever gotten into trouble driving because of drinking – like having an accident or being arrested for drunk driving?
Harm	Have you ever been arrested or held at the police station because of drinking or for disturbing the peace while driving?
Harm	Have you ever gotten into physical fights while drinking?
Harm	Have you ever had blackouts while driving, that is, where you drank enough so that you could not remember the next day what you had said or done?
Harm	Did drinking ever cause you to have liver disease or yellow jaundice?
Harm	Did drinking ever cause you to have vomiting blood or other stomach troubles?
Harm	Did drinking ever cause you to have trouble with tingling or numbness in your feet?
Harm	Did drinking ever cause you to have memory trouble when you haven’t been drinking (not blackouts)
Harm	Did drinking ever cause you to have inflammation of your pancreas, or pancreatitis?
Harm	Have you ever continued to drink when you knew you had a serious physical illness that might be made worse by drinking?
Dysfunction	Have you ever had “the shakes” after stopping or cutting down on drinking (for example, your hands shake so that your coffee cup rattles in the saucer or you have trouble lighting a cigarette)?
Dysfunction	Have you ever had fits or seizures after stopping or cutting down on drinking?
Dysfunction	Have you ever had the DT’s (Hallucinations and fever) when you quit drinking?
Dysfunction	Have you ever seen or heard things that weren’t really there after cutting down on drinking?
Dysfunction	Did you ever need a drink just after you had gotten up (that is, before breakfast)?
Dysfunction	Has there ever been a period in your life when you could not do your ordinary daily work well unless you had had something to drink?
Dysfunction	Have you ever wanted to stop drinking but couldn’t?

**Table A4:** **Harmful dysfunction (HD) diagnostic categories of harm and dysfunction and how they were translated using National Comorbidity Survey (NCS) questions**.

HD/NCS	HD/NCS (ECA comparable)	NCS alcohol question
Harm	Not used	Has your use of alcohol often kept you from working, going to school, or taking care of children?
Harm	Harm	Did alcohol ever cause you problems with your family, friends, at work, at school, or with the police?
Harm	Harm	Did your use of alcohol ever cause you to be expelled from school, or to be demoted or fired from work?
Harm	Harm	Have you often been under the effects of alcohol or feeling its after-effects in a situation which increased your chances of getting hurt – like when driving a car or boat, using knives or guns or machinery, crossing against traffic, climbing or swimming?
Harm	Harm	Did you continue to use alcohol after it caused an accident (when you injured yourself while under the influence of alcohol – like had a bad fall or cut yourself badly, been hurt in a traffic accident, or anything like that)?
Harm	Harm	Have you ever had any health problems as a result of using alcohol – such as liver disease, stomach disease, pancreatitis, feet tingling, numbness, memory problems, an accidental overdose, a persistent cough, a seizure of fit, hepatitis, or abscesses?
Harm	Harm	Have you ever had any emotional or psychological problems from using alcohol – such as feeling uninterested in things, feeling depressed, suspicious of people, paranoid, or having strange ideas?
Harm	Harm	Did you ever continue to use alcohol while taking medication you knew was dangerous to mix with alcohol or drugs, or when you had a serious health problem that could be made worse by alcohol or drugs?
Harm	Harm	Have you ever given up or greatly reduced important activities in order to get, or to use alcohol – activities like sports, work, or seeing family and friends?
Dysfunction	Dysfunction	Did stopping or cutting down on alcohol ever make you sick or cause you problems like those listed on page 17?
Dysfunction	Dysfunction	Did you ever use alcohol to make these withdrawal symptoms go away or to keep from having them?
Dysfunction	Dysfunction	Have you ever wanted or tried to stop or cut down on alcohol but found you could not?
Dysfunction	Not used	Have you ever felt such a strong desire or urge to use alcohol that you could not resist it or could not think of anything else?

**Table A5:** **DSM-5 diagnostic criteria for alcohol use disorder and their translation using National Comorbidity Survey (NCS) questions**.

DSM-5 Alcohol use disorder criteria	NCS questions used for the given criteria
A. A problematic pattern of alcohol use leading to clinically significant impairment or distress, as manifested by at least two of the following, occurring within a 12-month period	
1. Alcohol is often taken in larger amounts or over a longer period than was intended	Did you often use larger amounts of alcohol than you intended to when you began, or did you use it for a longer period of time than you intended to?
2. There is a persistent desire or unsuccessful efforts to cut down or control alcohol use	Have you ever wanted or tried to stop or cut down on alcohol but found you could not?
3. A great deal of time is spent in activities necessary to obtain alcohol, use alcohol, or recover from its effects	Did you ever have a period of a month or more when you spent a great deal of time using alcohol, getting it, or getting over its effects?
4. Craving, or a strong desire or urge to use alcohol	Have you ever felt such a strong desire or urge to use alcohol that you could not resist it or could not think of anything else?
5. Recurrent alcohol use resulting in a failure to fulfill major role obligations at work, school, or home	Has your use of alcohol often kept you from working, going to school, or taking care of children?
	Did your use of alcohol ever cause you to be expelled from school, or to be demoted or fired from work?
6. Continued alcohol use despite having persistent or recurrent social or interpersonal problems caused or exacerbated by the effects of alcohol	Did alcohol ever cause you problems with your family, friends, at work, at school or with the police?
	Did you continue to use alcohol after it caused an accident (when you injured yourself while under the influence of alcohol – like had a bad fall or cut yourself badly, been hurt in a traffic accident, or anything like that)?
7. Important social, occupational, or recreational activities are given up or reduced because of alcohol use	Have you ever given up or greatly reduced important activities in order to get, or to use alcohol – activities like sports, work, or seeing family and friends?
8. Recurrent alcohol use in situations in which it is physically hazardous	Have you often been under the effects of alcohol or feeling its after-effects in a situation which increased your chances of getting hurt – like when driving a car or boat, using knives or guns or machinery, crossing against traffic, climbing or swimming?
9. Alcohol use is continued despite knowledge of having a persistent or recurrent physical or psychological problem that is likely to have been caused or exacerbated by alcohol	Did you ever continue to use alcohol while taking medication you knew was dangerous to mix with alcohol or drugs, or when you had a serious health problem that could be made worse by alcohol or drugs?
10. Tolerance, as defined by either of the following:	Did you ever find that you had to use more alcohol than usual to get the same effect or that the same amount had less effect on you than before?
a. A need for markedly increased amounts of alcohol to achieve intoxication or desired effect	
b. Markedly diminished effect with continued use of the same amount of alcohol	
11. Withdrawal, as manifested by either of the following: a. The characteristic withdrawal syndrome for alcohol (refer to Criteria A and B of the criteria set for alcohol withdrawal)	Did stopping or cutting down on alcohol ever make you sick or cause you problems like those listed on page 17?
	Did you ever use alcohol to make these withdrawal symptoms go away or to keep from having them?
b. Alcohol (or a closely related substance, such as a benzodiazepine) is taken to relieve or avoid withdrawal symptoms	
