# Corrigendum: How many People have Alcohol Use Disorders? Using the Harmful Dysfunction Analysis to Rectify Prevalence Rates in Two Community Surveys

**DOI:** 10.3389/fpsyt.2014.00144

**Published:** 2014-10-16

**Authors:** Jerome C. Wakefield, Mark F. Schmitz

**Affiliations:** ^1^Silver School of Social Work and Department of Psychiatry, School of Medicine, New York University, New York, NY, USA; ^2^School of Social Work, Temple University, Philadelphia, PA, USA

**Keywords:** alcohol use disorder, alcohol dependence, addiction, validity of diagnosis, harmful dysfunction, diagnostic criteria, psychiatric epidemiology, National Comorbidity Survey

In this corrigendum, we identify and correct some misleading results concerning 1-year alcohol use disorder (AUD) reported in our earlier publication (1). The problems stem from coding anomalies in the epidemiological survey from which we drew data for our secondary analyses. Misinterpretations based on the misleading results are also corrected.

## The Problem

In our recent paper (1), guided by the harmful dysfunction (HD) analysis of mental disorder (2) and the impaired-control conception of AUD (3), we calculated new HD-type prevalence estimates for lifetime and 1-year AUD using data from two community surveys, the Epidemiological Catchment Area Survey (ECA) (4) and the National Comorbidity Study (NCS) (5). We compared rates across studies, and within the NCS, we compared rates yielded by different AUD definitions. These included two definitions we constructed for HD AUD and DSM-5 AUD, as well as standard NCS-defined variables for DSM-IV AUD (abuse or dependence) and DSM-IV dependence. We also assessed validity, unmet need, and remission based on these competing definitions.

A provocative finding in the NCS analysis was that the HD remission rate (defined as the percentage of those having lifetime AUD that do not also have 1-year AUD) was lower than remission rates derived using standard criteria. The finding of lower remission for HD-defined disorder potentially conflicted with the recent claim that AUD is not as persistent as traditionally thought (6, 7). This claimed lack of persistence has been the basis for questioning the nature of AUD and even whether it is a disorder at all.

In unpublished work, we attempted to replicate the NCS remission findings using another dataset, the National Epidemiologic Survey of Alcohol and Related Conditions (NESARC) (8). However, the lower HD remission rate was not replicated. HD remission was high and similar to remission rates using standard NESARC criteria.

## Source of the Problem

To identify the source of the cross-study discrepancy in HD AUD remission, we performed an item-level AUD-symptom persistence analysis for both the NESARC and NCS datasets, defining a symptom’s persistence as the percentage of individuals with the lifetime symptom that also had 1-year instances of the same symptom. For most symptoms, persistence was roughly 25–50%. Surprisingly, six NCS symptoms had 99 or 100% persistence rates, which made no sense (a seventh symptom implausibly persisted 0% of the time). Further exploration led us to identify problems in the original NCS coding of the six high-persistence symptoms. These symptom questions had been coded using exactly, or very nearly exactly, the same computer syntax for lifetime and 1-year symptoms, thus not allowing for validly distinguishing lifetime from 1-year symptoms. For those six symptoms, satisfying lifetime criteria virtually guaranteed also satisfying 1-year criteria even when the symptom had not in fact been experienced during the past year. The resulting inflation of 1-year rates due to the coding anomalies necessarily reduced remission rates. The six anomalously coded symptoms were disproportionately involved in HD criteria, yielding misleadingly low HD remission compared to standard criteria.

## Implications of Anomalous NCS 1-Year Criteria

Despite the anomalous 1-year NCS symptom measures, most of our earlier findings (1) remain valid because they concerned lifetime conditions, or report on standard NCS measures that we did not reconstruct, or they are otherwise independent of this particular issue. However, the anomalous coding did have potential consequences for 1-year AUD findings involving some claims about our own reconstructed criteria. Coding anomalies affected the prevalence of our constructed variables of 1-year HD AUD and 1-year DSM-5 AUD, as well as 1-year validity comparisons, unmet need estimates, and remission findings. We emphasize that the bulk of results reported in our original paper, including all lifetime AUD prevalence, validator, and unmet need analyses, remains valid.

## Reanalysis Strategy

We performed reanalyses testing whether our basic conclusions and results were maintained when coding problems were corrected. We modified only our constructed HD and DSM-5 AUD criteria. As in our original article, we did not attempt to reconstruct standard NCS variables, including the NCS standard 1-year AUD that includes abuse and dependence with at least one symptom in the last year, NCS narrow 1-year AUD that requires all symptoms occur in the past year, and NCS dependence [see Ref. (1) for fuller descriptions of criteria]. We continue to use these standard NCS variables as comparison baselines in evaluating HD criteria. However, the coding problems we uncovered do suggest that standard NCS 1-year estimates are ultimately problematic.

We first recoded all NCS 1-year alcohol symptoms used in the HD and DSM-5 analyses, simplifying the coding structure so that they directly assessed the issue of whether the specific symptom occurred during the past year. For five of the seven problematic symptoms, this resolved the problem; testing revealed plausible persistence levels in the same range as other symptoms. To be consistent, we applied the same simplified structure to all items used in the criteria. We found that this alteration did not affect the persistence of the non-problematic symptoms.

However, for two problematic symptoms, the NCS questionnaire did not ask about the last occurrence, which was the basis for our past-year assessment. The items were: “Did you continue to use alcohol after you had accidentally injured yourself while under the influence of alcohol?” and “Did you use alcohol to make these withdrawal symptoms go away or to keep from having them?” Thus, there was no way to reconstruct the criteria to distinguish 1-year from lifetime occurrence of these symptoms. Both of these symptoms were components of our HD AUD criteria, in which the “injury” symptom is a harm and the “prevent withdrawal” symptom is a dysfunction. Thus, an individual having these two lifetime symptoms and no 1-year symptoms would be mistakenly classified as having 1-year HD disorder. The same problem afflicted our DSM-5 criteria, which also required two symptoms for AUD diagnosis. The “injury” item satisfied DSM-5’s “continued use” criterion and the “prevent withdrawal” item satisfied DSM-5’s “withdrawal” criterion, thus allowing 1-year DSM-5 AUD to be diagnosed on the basis of the two lifetime symptoms alone.

So, second, we adjusted the HD and DSM-5 1-year criteria so as to remain as close as possible to the original HD and DSM-5 AUD categories while ensuring that 1-year disorder always involved at least one valid 1-year symptom. For HD, we adjusted the 1-year criteria to allow the lifetime “injury” symptom to qualify as 1-year harm only if there was also a 1-year dysfunction other than “prevent withdrawal,” and similarly we allowed “prevent withdrawal” to be a qualifying 1-year dysfunction only if there was a 1-year harm other than “injury.” We similarly adjusted DSM-5 criteria so that an individual could not be diagnosed with 1-year disorder on the basis of the two lifetime symptoms alone, but must have at least one explicitly 1-year symptom for 1-year diagnosis. We used these modified HD and DSM-5 criteria in the reanalyses reported below.

## Results of Reanalyses

Demographic characteristics of the two modified groups did not change from previously published values (1). Revised prevalence, validator, and unmet need estimates using the modified HD and DSM-5 1-year AUD criteria are shown in Table 1, including comparisons to results using NCS standard criteria reported in the original paper.

**Table 1:** **One-year prevalence, 1-year unmet need, and means and percentages (95% confidence intervals) of five validators of 1-year alcohol use disorder (AUD), compared for five definitions of AUD: National Comorbidity Study (NCS) standard, NCS narrow, NCS dependence, HD, and DSM-5 AUD**.

	NCS standard	NCS narrow	NCS	Modified	Modified
	AUD	AUD	dependence	HD AUD	DSM-5 AUD
	1-year	1-year	1-year	1-year	1-year
	(*n* = 793)	(*n* = 539)	(*n* = 597)	(*n* = 213)	(*n* = 579)
Prevalence (% US population)	9.9 (8.9, 11.0)	7.0 (6.1, 7.9)	7.4 (6.5, 8.4)	2.7 (2.1, 3.3)	7.3 (6.5, 8.0)
Unmet need (% US population)	7.4 (6.5, 8.4) [*n* = 577]	5.1 (4.4, 5.8) [*n* = 381]	5.1 (4.3, 5.9) [*n* = 399]	1.3 (0.9, 1.7) [*n* = 101]	5.2 (4.4, 5.9) [*n* = 396]
Mean duration, years	10.4^a^ (9.3, 11.4)	10.5^b^ (9.3, 11.7)	10.5^c^ (9.3, 11.7)	12.9^a,b,c,d^ (11.2, 14.6)	10.1^d^ (8.9, 11.4)
% See mental health professional about substance use, ever	11.9 (9.1, 14.8)	11.6 (8.7, 14.4)	14.7^a^ (11.2 18.1)	24.1^a^ (18.1, 30.0)	13.4 (10.2, 16.7)
% Attended AA or NA meetings, ever	20.5 (16.1, 24.9)	23.3 (18.0, 28.6)	25.7 (19.8, 31.7)	47.9 (39.1, 56.8)	24.4 (18.4, 30.3)
% Went to drug or alcohol outpatient clinic, ever	7.8 (5.3, 10.4)	8.5 (5.1, 12.0)	9.7^a^ (6.6, 12.8)	19.5^a,b^ (12.2, 26.8)	9.4^b^ (5.9, 13.0)
% Have any NCS mood or anxiety disorder, lifetime	50.1^a^ (44.4, 55.8)	49.9^b^ (43.2, 56.6)	53.9 (48.0, 59.8)	61.2^a,b,c^ (51.1, 71.3)	53.4^c^ (46.4, 60.4)

*^a,b,c,d^Significant differences, using Wald *F*-test*.

*Ages 18–54, *N* = 7,599, NCS data. Weighted and corrected for sampling design. Using modified 1-year alcohol symptom criteria for HD and DSM-5 analyses*.

As expected, the HD and DSM-5 1-year prevalence estimates using the modified criteria are considerably lower than the earlier values (1). HD prevalence remains significantly and substantially below other estimates, decreasing from 4.3% to the modified rate of 2.7%. DSM-5 decreased from 9.8% to the modified rate of 7.3%.

Despite the changed prevalences, validator levels and results of validator comparisons remained roughly the same. When we calculated the modified categories’ validator levels, in no case was the modified 1-year HD or DSM-5 validator level significantly different from the previously published level. The results of the comparison of validator levels across AUD definitions also remained essentially the same as previously published. Across all five of our validators (duration, three service use indicators, and comorbidity), 1-year HD AUD continued to show significantly greater pathology levels than 1-year standard NCS AUD, standard NCS narrowly-defined AUD, standard NCS dependence, and modified DSM-5 AUD, with one exception; 1-year HD AUD is no longer significantly higher than 1-year NCS dependence in comorbid mood or anxiety disorders. With lower prevalence and high service use rates, HD 1-year unmet need decreased from the already low 1.8% to the modified rate of 1.3% of the adult population. DSM-5 1-year unmet need decreased from 6.3 to 5.2%.

## Modified Remission Results

The original remission analysis (1) indicated a lower remission rate for HD AUD (37%) than for standard NCS (60%) or DSM-5 (50%) AUD. This, we observed, appeared to suggest that tighter criteria may yield lower remission. However, these results and implications are disconfirmed by the modified analysis reported here. The modified HD remission rate (60%) is about the same as the standard NCS rate (60%) and the modified DSM-5 rate (58%). Contrary to earlier claims, more conceptually valid HD criteria with higher validator levels do not yield lower remission. The broader implications of these corrected results must await future discussion.

## Conclusion

Problems identified in NCS coding of 1-year symptoms were corrected in modified analyses. The modified results support all of the conclusions and interpretations regarding prevalence comparisons, validator levels, and unmet need reported in our original analysis (1), with one important exception. HD AUD does not have lower remission rates than standardly measured AUD.

## Conflict of Interest Statement

The authors declare that the research was conducted in the absence of any commercial or financial relationships that could be construed as a potential conflict of interest.
